# A physics-informed LLM agent for uncovering typhoon- and earthquake-induced turbine failures and offline yaw-pitch countermeasures

**DOI:** 10.1016/j.isci.2026.118076

**Published:** 2026-10-30

**Authors:** Linjun Fu, Zhipeng Xie, Shaohai Zhang, Lilin Wang, Lizhong Wang, Huajun Chen

**Affiliations:** 1 Ocean College & State Key Laboratory of Ocean Sensing, Zhejiang University, Zhoushan 316021, China; 2 Zhejiang Province Key Laboratory of Offshore Civil Engineering and Materials & Ocean Academy, Zhejiang University, Zhoushan 316021, P.R. China; 3 College of Computer Science and Technology, Zhejiang University, Hangzhou 310058, P.R. China; 4 Institute of Fundamental and Transdisciplinary Research, Zhejiang University, Hangzhou 310058, P.R. China; 5 Shanghai Institute for Advanced Study, Zhejiang University, Hangzhou 310058, P.R. China; 6 School of Computer Science, East China Normal University, Shanghai 200241, China

**Keywords:** wind turbine dynamics, typhoon and earthquake loading, large language model agents, Zwind, automated load simulation, model context protocol

## Abstract

Extreme typhoons and earthquakes can cause severe damage to wind turbine structures, underscoring the need to better understand failure mechanisms and potential mitigation strategies. This study develops Iwind, a physics-informed large language model agent for wind-turbine engineering analysis. Supported by domain-specific training and multi-path retrieval-augmented generation, Iwind combines ReAct-style reasoning with the model context protocol to invoke the in-house physics solver Zwind for natural-language-driven load simulations. In the reported evaluations, Iwind outperformed its parent model on wind-turbine knowledge tasks and supported load-simulation execution and visual damage inspection. The collected cases showed blade fractures, tower buckling, and overturning under typhoon loading, while some earthquake-damaged turbines showed more pronounced damage in foundations or lower supporting structures. Parametric Zwind simulations further revealed W- and M-shaped offline yaw-pitch response manifolds for the investigated typhoon and earthquake cases, respectively, providing simulation-derived references for parked-state comparison and hazard-region identification.

**Video Abstract:**

## Highlights

• A physics-informed language-model agent automates wind-turbine simulation workflows
• Domain adaptation improves benchmark and end-to-end simulation-task performance
• Typhoon and earthquake cases show distinct wind-turbine failure patterns
• Offline yaw-pitch manifolds support parked-state comparisons under extreme loads

## Graphical abstract

**Figure:**
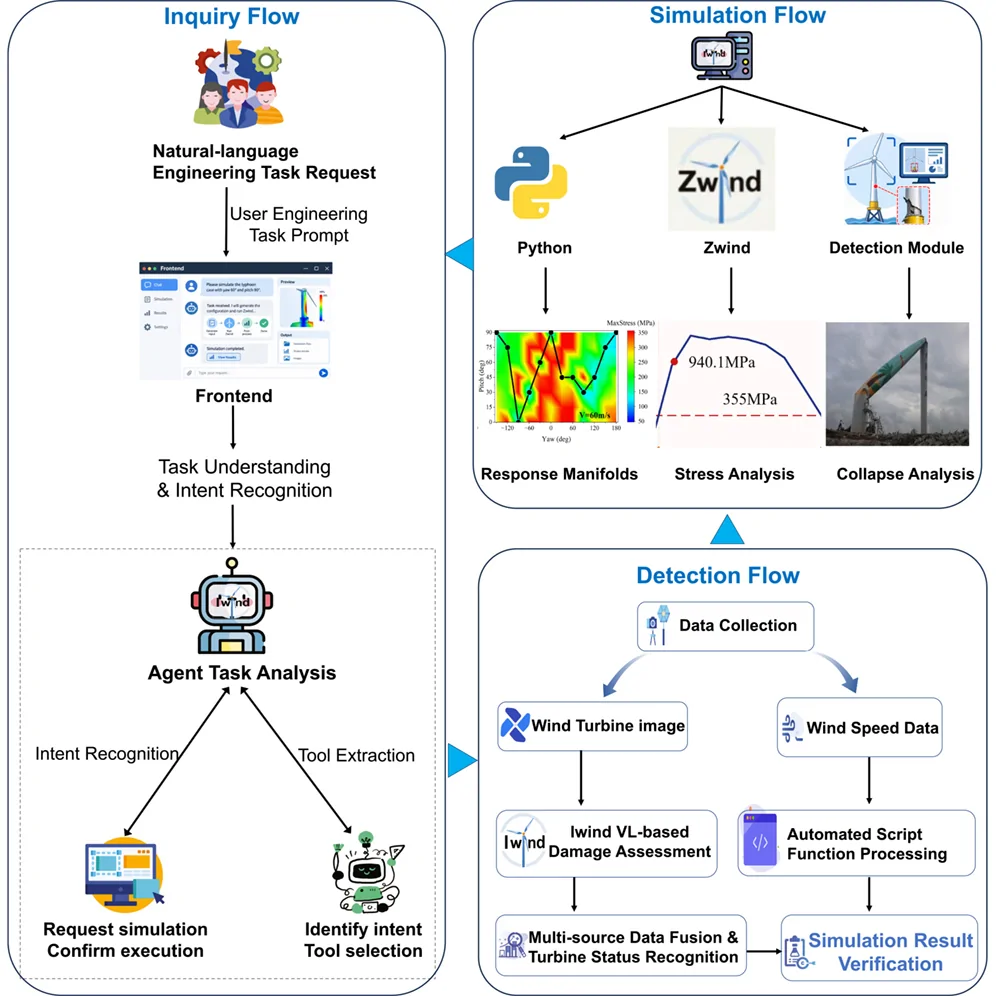

## Teaser

Artificial intelligence; Computing methodology; Energy resources; Energy sustainability; Physics

## Introduction

With 117 GW installed in 2024, wind power is advancing into new geographies and consolidating its position as a core pillar of the global energy transition.^1^ As the world’s largest regional wind energy market in terms of both cumulative and newly installed capacity,^2^ China lies at the forefront of this expansion. However, this vast infrastructure faces unprecedented risks from extreme hazards, particularly typhoon and earthquake. For instance, an average of nine typhoons make landfall along China’s southeastern coast annually.^3^ Despite frequent reports of structural failures following extreme environmental loading, quantitative investigations, and reliable design countermeasures remain scarce.

Historically, uncovering the failure mechanisms of wind turbines under extreme typhoons has remained an open challenge. In September 2003, Typhoon Maemi struck Japan with estimated peak gusts reaching 90 m/s. During this catastrophe, Ishihara et al.^4^ investigated the collapse of multiple turbine towers and revealed that the typhoon-induced maximum bending moments significantly exceeded the towers’ ultimate capacities. Similarly, during Typhoon Jangmi in Taiwan in 2008, Chou et al.^5^^,^^6^ attributed tower collapses and blade damages to insufficient material strength, resonance effects, and control failures. Several years later, Super Typhoon Usagi (2013) devastated a coastal wind farm in Guangdong Province,^7^ destroying 35 blades and causing approximately 16 million RMB in direct economic losses. Most recently, Super Typhoon Yagi (2024) demonstrated that even in an era of globally advanced technology, turbines in regions like Wenchang, Hainan, still experience widespread structural destruction.^8^ These consecutive events indicate that the failure mechanisms under high wind speeds and large yaw angles are still not fully understood.

In addition to extreme wind loads, recent installations of numerous wind turbines in earthquake-prone areas worldwide (e.g., China, the USA, India, and East Asia) highlight the necessity for thorough consideration of the seismic implications on these energy-harnessing systems. While researchers have increasingly recognized the importance of seismic loads on operational wind turbines in recent years, comprehensive studies remain limited. Most existing literature focuses on identifying key factors that affect the seismic responses of wind turbine structures (Witcher^9^; Ishihara and Sawar^10^; Asareh et al.^11^; Valamanesh and Myers^12^). Witcher^9^ examined parked, operational, and earthquake-induced emergency-shutdown cases for a 2 MW wind turbine using the GH Bladed^13^ seismic module. Asareh et al.^11^ evaluated the coupled aerodynamic and seismic responses of the NREL 5 MW reference turbine under multiple operational scenarios. Ishihara et al.^14^ subsequently showed that the governing operational scenario depends on the misalignment between the wind and earthquake directions: emergency shutdown produced the maximum tower-base moment at 0° misalignment, whereas normal operation produced the maximum load at 90° misalignment. This finding highlights the importance of directional wind-seismic coupling.

Despite these foundational efforts, systematic evidence on structural failure patterns under extreme earthquake events and corresponding engineering countermeasures remains limited.

Traditionally, investigating these extreme typhoon and seismic failures relies heavily on tedious manual field surveys and isolated numerical simulations. As disaster assessment tasks grow increasingly complex and labor-intensive, researchers are increasingly turning to artificial intelligence (AI) as a route to workflow automation. A recent review highlights how machine learning can support the analysis and interpretation of extreme events.^15^ For instance, AI-assisted tools have demonstrated potential in automating parts of extreme weather assessment, including forecasting support and risk communication.^16^ Aurora exemplifies this trend as a large-scale foundation model for Earth system forecasting, and it achieves state-of-the-art performance in tasks such as weather forecasting and air-quality prediction.^17^ By generating rapid predictions from massive datasets, these data-driven models substantially strengthen early-warning systems for natural disasters.^18^^,^^19^

Recent studies suggest that AI-enabled agents can streamline specific parts of scientific workflows across various domains. For example, DisasTeller^20^ is a framework powered by multiple large vision-language models (LVLMs) designed to automate post-disaster management tasks, including on-site assessment, emergency alerts, resource allocation, and recovery planning. By coordinating four specialized LVLM agents with GPT-4 as the core, DisasTeller accelerates disaster response activities, reduces manual execution time, and structures information flow, although it still requires human validation and further improvements in LVLM accuracy. Despite these advances, DisasTeller does not link visual assessments to physics-based simulations, nor does it form a closed engineering analysis loop. Similar agents have been devised in other areas. For instance, SciToolAgent presents a large-language-model agent that uses a scientific tool knowledge graph for tool selection and multi-tool orchestration across scientific tasks.^21^ In the biochemical domain, PLMeAE introduces an automatic evolution platform enabled by protein language models that leverages protein language models for learning and design, executing build-and-test stages within a closed-loop automated biofoundry.^22^ In marine science, OceanGPT proposes a domain-specific large language model and a multi-agent framework to generate ocean-domain instructional data and evaluation benchmarks.^23^ While these existing advances successfully automate knowledge-level processes, such as information acquisition, organization, and reasoning, they generally fall short of automating the execution of engineering-level simulations. In complex engineering scenarios such as typhoon- and earthquake-induced damage investigations presented in this study, automated simulation combined with comparison with available observations remains essential for efficient and reliable analysis.^19^

Focusing on wind turbine structures under extreme typhoon and earthquake loading, this study establishes an integrated physics-simulation-driven LLM agent for wind-turbine engineering. Its central contribution is to connect natural-language engineering requests with executable and traceable simulation workflows. Within this framework, the Iwind agent performs intent recognition, task decomposition, simulation orchestration, MCP-based tool invocation, result parsing, and closed-loop consistency checking, whereas Zwind performs the underlying physics-based calculations. This division supports the systematic analysis of structural responses, damage mechanisms, and candidate parked-state control guidance under the investigated typhoon and earthquake conditions. Domain-specific model training, multi-path RAG, MCP-based solver interfacing, and external visual-recognition modules were adapted and integrated to support the natural-language-driven engineering workflow, with the corresponding training, retrieval, tool-execution, and visual-analysis procedures detailed in STAR Methods.

## Results

### Architecture of the Iwind agent

Figure 1 presents the five functional modules through which the Iwind agent organizes physics-informed task execution.^24^^,^^25^

**Figure 1:**
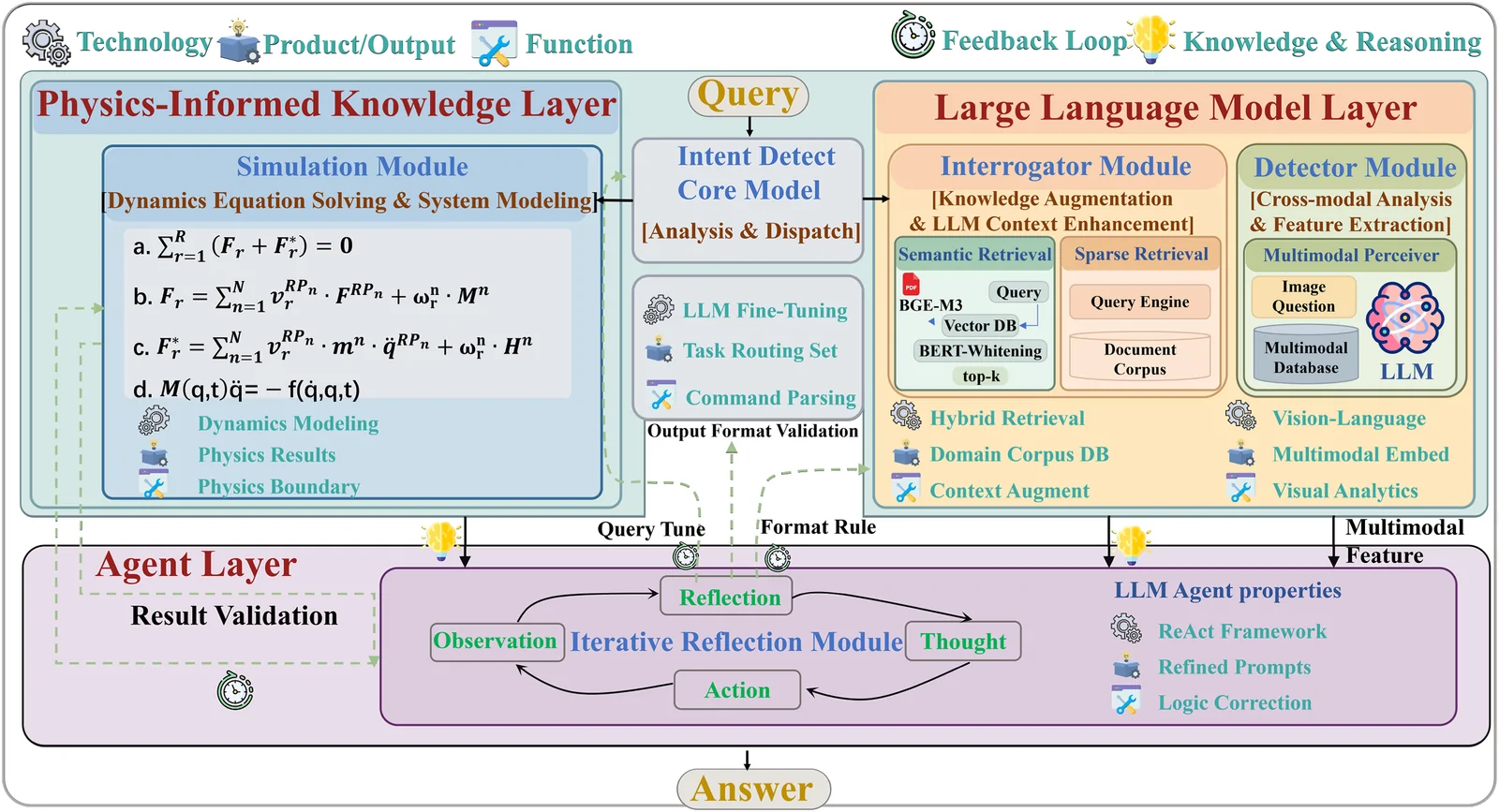
Architecture of the Iwind agent Zwind serves as the physics solver within the simulation module.

The intent recognition module (the recognizer) interprets user queries and task objectives, while the iterative reflection module (the reflector) improves system performance through continuous feedback and iterative refinement. The knowledge reasoning module, referred to as the Interrogator, employs retrieval-augmented generation (RAG)^26^^,^^27^^,^^28^ with multi-path retrieval to provide structured domain knowledge support for typhoon or earthquake damage analysis of wind turbine structures. The simulation execution module, referred to as the simulator, uses the model context protocol (MCP) as a standardized interface^29^^,^^30^ to integrate the proprietary wind turbine CAE software Zwind, thereby enabling complex loading and structural response simulations for wind turbine structures. The perception module, referred to as the detector, combines computer vision and visual language models to process multimodal inputs and to enable automated damage detection and fault localization. Through coordinated interactions among these modules, the Iwind agent converts engineering requests into simulation tasks, prepares the required inputs, invokes Zwind and the post-processing scripts, and organizes the resulting evidence for analysis and reporting. Zwind performs the coupled physical calculations and generates the corresponding loads and structural responses. This integrated design improves the traceability and efficiency of typhoon- and earthquake-related damage assessment.

### From base model to agent tasks

We evaluated five configurations derived from the same DeepSeek-R1-0528-Qwen3-8B parent model on WindBench, an independent benchmark containing 15,000 Chinese question-answer pairs across 15 task categories. Table 1 reports their absolute scores.

**Table 1:** Performance of five configurations based on the same DeepSeek-R1-0528-Qwen3-8B parent model on the Chinese WindBench benchmark

Model configuration	BLEU-4	ROUGE-1	ROUGE-2	ROUGE-L	Text coverage
Base model	0.20	21.9	9.2	20.9	20.0
Base model + RAG	0.35	27.8	12.1	25.9	29.5
SFT model	0.72	34.6	16.2	32.4	32.8
SFT + RL model	0.93	38.7	18.7	36.1	35.6
Full model: SFT + RL + RAG	1.10	41.5	20.4	38.9	40.0

Comparison of five model configurations derived from the same parent model on WindBench using BLEU-4, ROUGE-1, ROUGE-2, ROUGE-L, and Text Coverage metrics.

All metrics are reported as absolute scores on a 0–100 scale and were calculated on the same 15,000 Chinese question-answer pairs. Each pair has one expert-reviewed reference answer. Table 1 shows staged score increases across the evaluated configurations. The base model + RAG configuration obtained higher scores than the base model, while supervised fine-tuning produced further gains in ROUGE and text coverage. Adding RL to the SFT model increased all five reported scores. The full configuration achieved the highest values in this comparison, reaching 1.10 for BLEU-4, 41.5 for ROUGE-1, 20.4 for ROUGE-2, 38.9 for ROUGE-L, and 40.0 for text coverage.

The absolute BLEU-4 scores remained low. WindBench includes open-ended technical questions and provides only one expert-reviewed reference answer for each item. Semantically acceptable responses expressed using different wording can therefore obtain limited surface-form 4-g overlap. BLEU-4 is thus treated as an auxiliary surface-matching metric and is interpreted alongside ROUGE and text coverage.

To assess practical relevance, we applied Iwind agent to three representative offshore wind power tasks such as popular science QA, load simulation, and visual damage detection.

Figure 2 compares responses of the base model and Iwind agent to two representative questions. In these examples, the Iwind agent generated more structured and domain-specific responses, including turbine components, life cycle considerations, and several deep-sea wind-power development directions.

**Figure 2:**
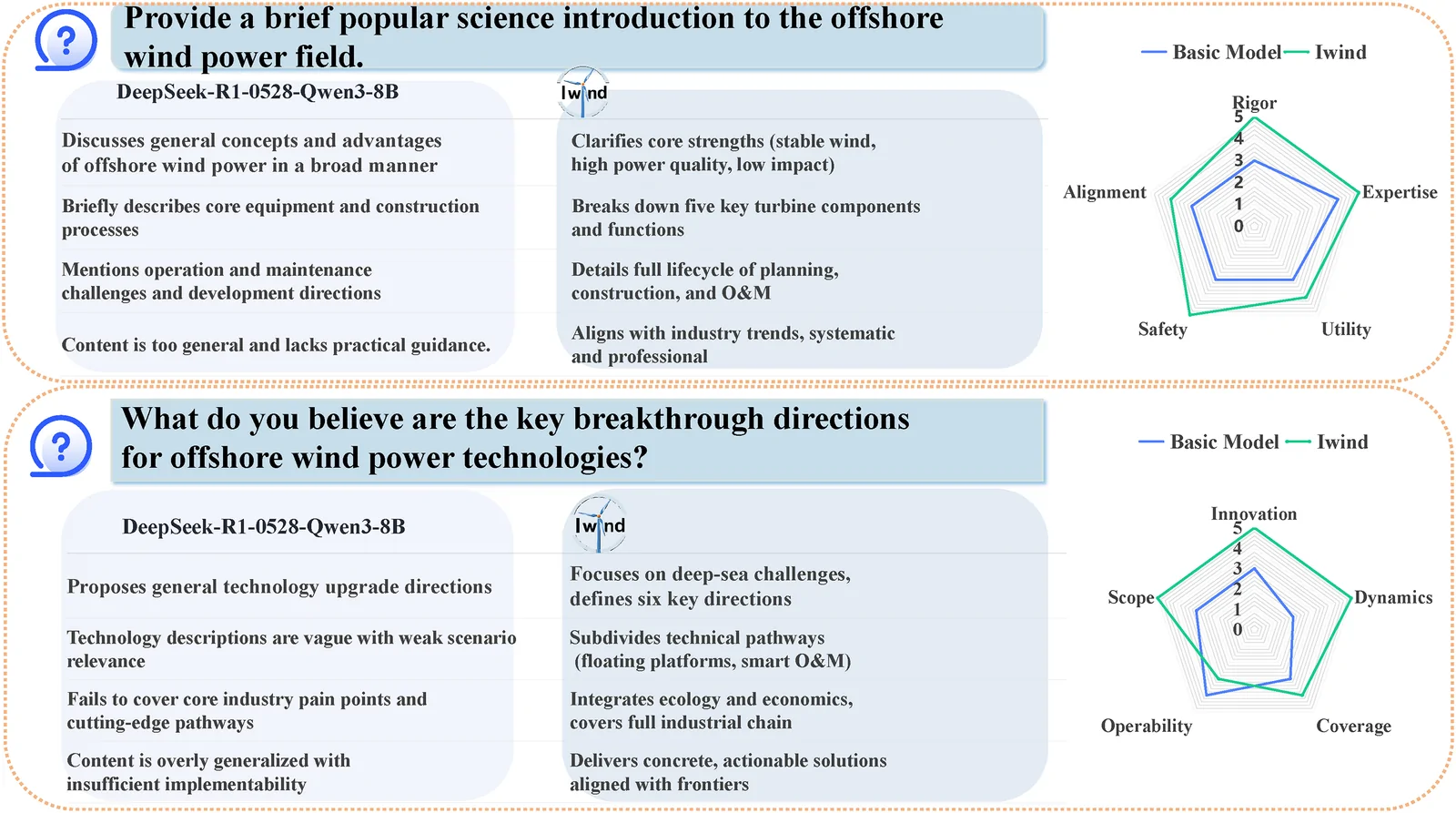
Response comparison: base model vs. Iwind agent Iwind is used as an abbreviated label for the Iwind agent.

Beyond question answering, a second core capability of the Iwind agent is the natural-language-driven automation of Zwind load simulations. Starting from a user request, the agent interprets the task, extracts and checks the required parameters, generates Zwind-compatible input files, invokes the solver through MCP, and collects the resulting output folders and files. Zwind, rather than the agent, performs the corresponding numerical calculations under the prescribed model assumptions. Once the simulation is completed, deterministic post-processing scripts are invoked to extract and organize the results for visualization, engineering interpretation, and reporting. The publicly available code and reproducibility materials implement this workflow, allowing readers to independently execute and verify the central process from natural-language input to simulation output and deterministic post-processing.

The simulation-input generation and end-to-end task-completion results in Table 2 provide a separate evaluation of the model’s ability to execute engineering tasks.

**Table 2:** Ablation results for Zwind simulation-input generation and end-to-end tool-use tasks

Model configuration	Simulation input generation	End-to-end task completion
Base model	19/30 (63.3%)	18/30 (60.0%)
Base model + RAG	22/30 (73.3%)	21/30 (70.0%)
SFT model	22/30 (73.3%)	22/30 (73.3%)
SFT + RL model	24/30 (80.0%)	23/30 (76.7%)
Full model: SFT + RL + RAG	26/30 (86.7%)	25/30 (83.3%)

Evaluation of the incremental effects of different model configurations on automated Zwind simulation-input generation and complete tool-use workflow execution.

To evaluate the agent’s ability to operate the Zwind toolchain independently of the 15-category WindBench text benchmark, we conducted a controlled ablation study using the same 30 predefined simulation tasks for each model configuration. All configurations used the same parent model, simulation tasks, decoding parameters, MCP tool definitions, and Zwind simulation environment. Each trial was evaluated using a binary success-or-failure criterion.

Table 2 evaluates two workflow-level capabilities: simulation input generation and end-to-end task completion. A simulation-input generation trial was considered successful only when the generated file conformed to the required Zwind format without manual modification, contained all task-specific required parameters consistent with the corresponding reference input, and could be read by Zwind to initiate the calculation. An end-to-end task was considered successful only when the agent completed intent recognition, parameter extraction, simulation-input generation, MCP-based tool invocation, simulation execution, generation of the expected output folder and .out file, maximum-stress extraction, and final engineering reporting. In addition, the absolute difference between the maximum stress obtained from the automated workflow and that obtained from the manually configured Zwind simulation for the same case was required to be no greater than 5 MPa. This threshold was used as an operational consistency criterion for the workflow comparison and not as an independent validation of the physical accuracy of Zwind.

As shown in Table 2, both domain adaptation and retrieval augmentation improved the execution of Zwind-related tasks relative to the base model. Adding RL to the SFT model further increased the simulation-input generation rate from 73.3% to 80.0% and the end-to-end task completion rate from 73.3% to 76.7%. The full configuration achieved the highest rates, reaching 86.7% for simulation-input generation and 83.3% for end-to-end completion. These staged comparisons indicate complementary incremental benefits from SFT, RL, and RAG within the evaluated workflow. Because not all possible component combinations were tested, the results should not be interpreted as a complete factorial analysis of independent main effects or interactions. MCP, ReAct, RAG, LoRA, and GRPO remain established enabling methods; the evaluated contribution lies in their domain-specific use together with the offshore-wind corpus, WindBench, and the executable coupling between the Iwind agent and Zwind.

These controlled comparisons were intended to assess the incremental effects of SFT, RL, and RAG under a fixed parent model and workflow, rather than to establish a cross-model ranking against general-purpose frontier models.

For visual damage detection, Iwind agent enabled an end-to-end pipeline from turbine component images to maintenance recommendations. The visual pipeline screened the input images for seven visible surface-defect categories (burn, crack, deformation, dirt, oil stain, peeling, and rust) and used the detected regions as inputs for subsequent severity assessment and generation of maintenance suggestions. The YOLO detector was evaluated on the held-out test set. The best-performing checkpoint achieved a precision of 0.615, a recall of 0.427, an mAP@0.5 of 0.482, and an mAP@0.5:0.95 of 0.221, as shown in Table 3. The maximum overall F1 score was 0.490 at a confidence threshold of 0.322. The class-specific AP@0.5 values ranged from 0.232 to 0.715, indicating moderate and class-dependent performance. Accordingly, this study uses the detector as an auxiliary module for visible surface-defect screening rather than as a standalone tool for structural-damage diagnosis.

**Table 3:** Overall and class-specific performance of the YOLO detector on the held-out test set

Overall metric	Value	Defect class	AP@0.5
Precision	0.615	Burn	0.715
Recall	0.427	Crack	0.383
mAP@0.5	0.482	Deformation	0.456
mAP@0.5:0.95	0.221	Dirt	0.617
F1	0.490	Oil stain	0.232
Confidence at best F1	0.322	Peeling	0.425
–	–	Rust	0.543

The 5,000-image dataset was divided into training, validation, and held-out test sets at a ratio of 7:2:1, corresponding to 3,500, 1,000, and 500 images, respectively. The reported values were obtained using the best-performing checkpoint. AP denotes average precision. Values are rounded to three decimal places.

Summary of overall detection performance and class-specific average precision for seven visible wind-turbine surface-defect categories on the held-out test set.

### Typhoon damage assessment

Figure 3 illustrates the Iwind agent as a fully integrated closed-loop workflow that begins with typhoon damage perception and advances through information deduction, simulation execution, and simulation and observation recheck.

**Figure 3:**
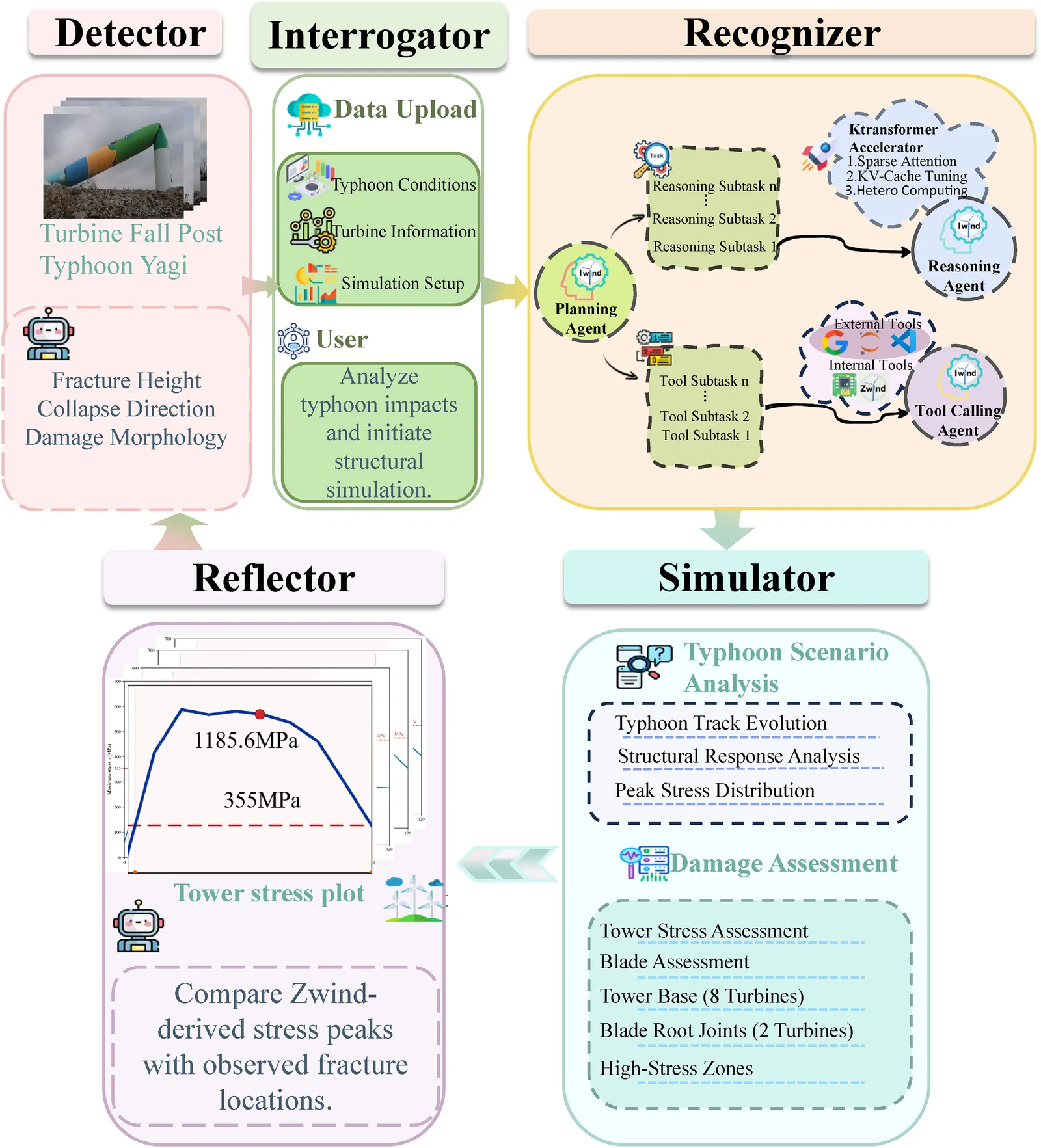
Flowchart of typhoon damage analysis The workflow connects typhoon damage perception, information deduction, simulation execution, and simulation-observation recheck through the detector, interrogator, recognizer, simulator, and reflector modules.

This streamlined workflow enables smooth data flow and interaction across all stages. Initially, multi-source field data are processed to derive key typhoon characteristics, including strength, trajectory, and impact zone. In parallel, wind turbine-relevant parameters, such as structural integrity, design specifications, and operational conditions, are incorporated into the system. To establish the observational baseline, damage evidence was systematically gathered from field photography and publicly available online sources, including meteorological records and satellite images. The detector utilizes advanced image processing techniques to extract structured attributes from these images, focusing on key features such as fracture height, collapse direction, and visible damage morphology. Figure 4 summarizes the spatial distribution and image-derived damage observations for the 15 turbines considered in the Typhoon Yagi case.

**Figure 4:**
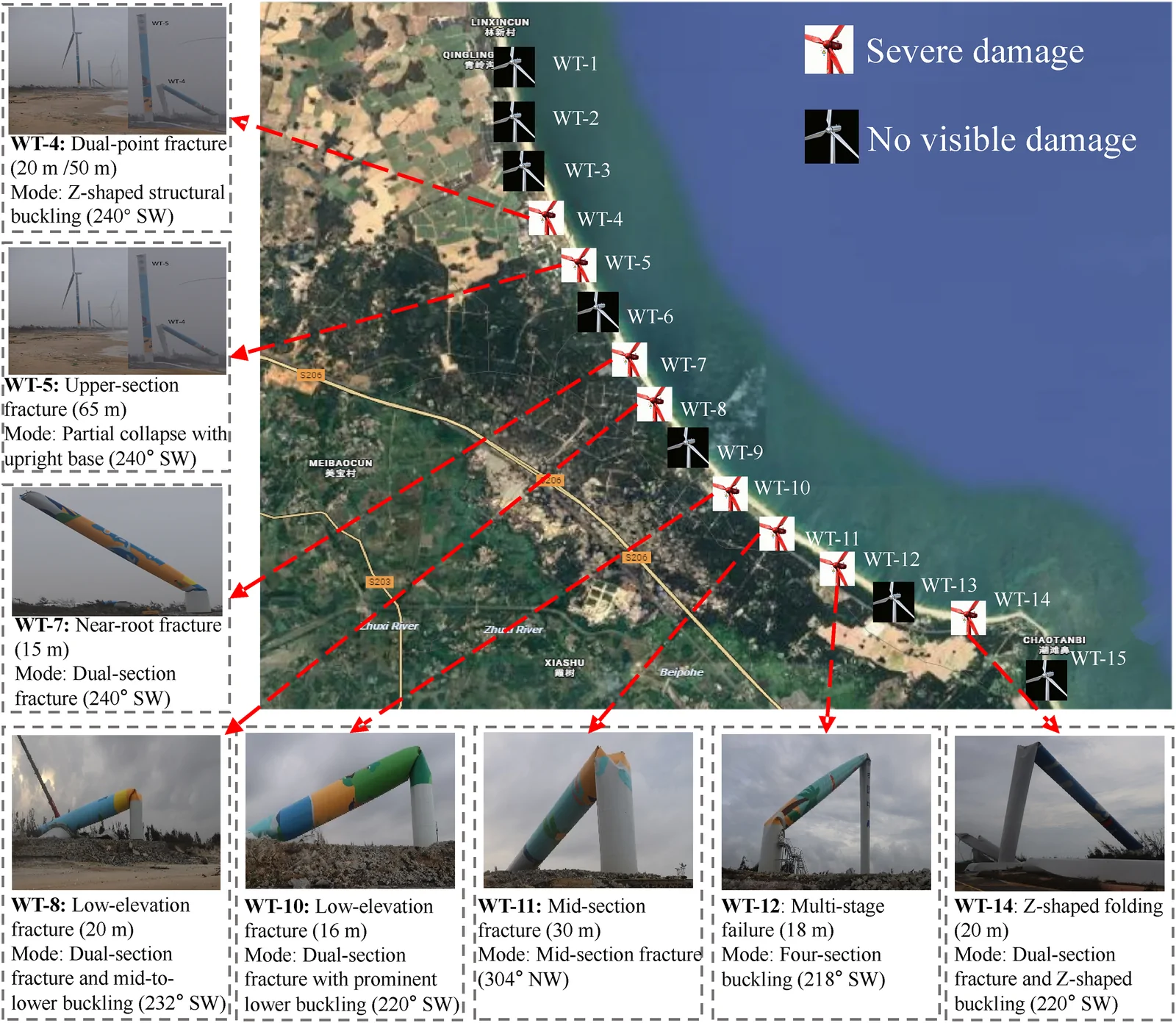
Typhoon damage observations for the 15 turbines considered in this study The “No visible damage” label indicates that no obvious external damage was identified in the available images; it does not exclude hidden or internal structural damage.

These observations indicate that the turbines sustained substantial visible structural damage under typhoon loading, particularly along the direction of the typhoon trajectory. Among the 15 turbines analyzed in this study, eight exhibited tower collapse. As illustrated in Figure 4, the estimated fracture heights of the collapsed towers were predominantly concentrated between 15 and 65 m, while their collapse directions generally aligned with the downwind direction of the typhoon. The image-based assessment further identified several visible damage patterns, including two-stage tower fractures, C-shaped localized deformation, and fractures in the upper portions of the towers. Height-dependent wind loading, including wind shear and turbulence, provides a possible explanation for the observed upper-level fracture locations.

The fracture heights reported in Figure 4 are approximate values estimated by the vision module from the collected post-event images. The C- and Z-shaped labels describe the visible post-event damage morphologies identified from these images. By contrast, the “No visible damage” label assigned to WT-1, WT-2, WT-3, WT-6, WT-9, WT-13, and WT-15 indicates only that no obvious external damage was identified in the available images.

To qualitatively relate the image-based damage observations to the simulated structural responses, the corresponding environmental conditions and turbine orientations were used to configure the high-fidelity Zwind simulation model (the Simulator) for structural dynamic analysis. The simulations were driven by a typhoon wind field reconstructed using the Holland model with track and intensity parameters obtained from the China Meteorological Administration (CMA) Tropical Cyclone Best Track Dataset.^31^ For the typhoon event examined in this study, the reconstructed wind field yielded a maximum wind speed of 60 m/s, and this model-derived value was subsequently used in the structural simulations. Wind direction and nacelle yaw angle are jointly specified for each turbine according to its orientation relative to the incoming flow. Eight wind turbines are simulated, covering azimuthal orientations ranging from 0° to 180°. Geometric transformation is applied to obtain the effective wind direction acting on each turbine, and the corresponding nacelle yaw angle is assigned accordingly. The total simulation duration is 3,600 s, with a computational time step of 0.005 s and an output interval of 0.05 s, providing sufficient temporal resolution for capturing tower dynamic responses. Zwind calculated the linear-elastic sectional stress demand along the tower height. The approximate fracture heights estimated from the post-event images were mapped onto the corresponding stress profiles, and the calculated stresses at these heights are marked by red dots in Figure 5. The red dashed line denotes the 355 MPa yield-strength threshold of the S355 tower steel and is used to identify yield-exceedance regions. Figure 5 summarizes this comparison for the eight damaged turbine towers.

**Figure 5:**
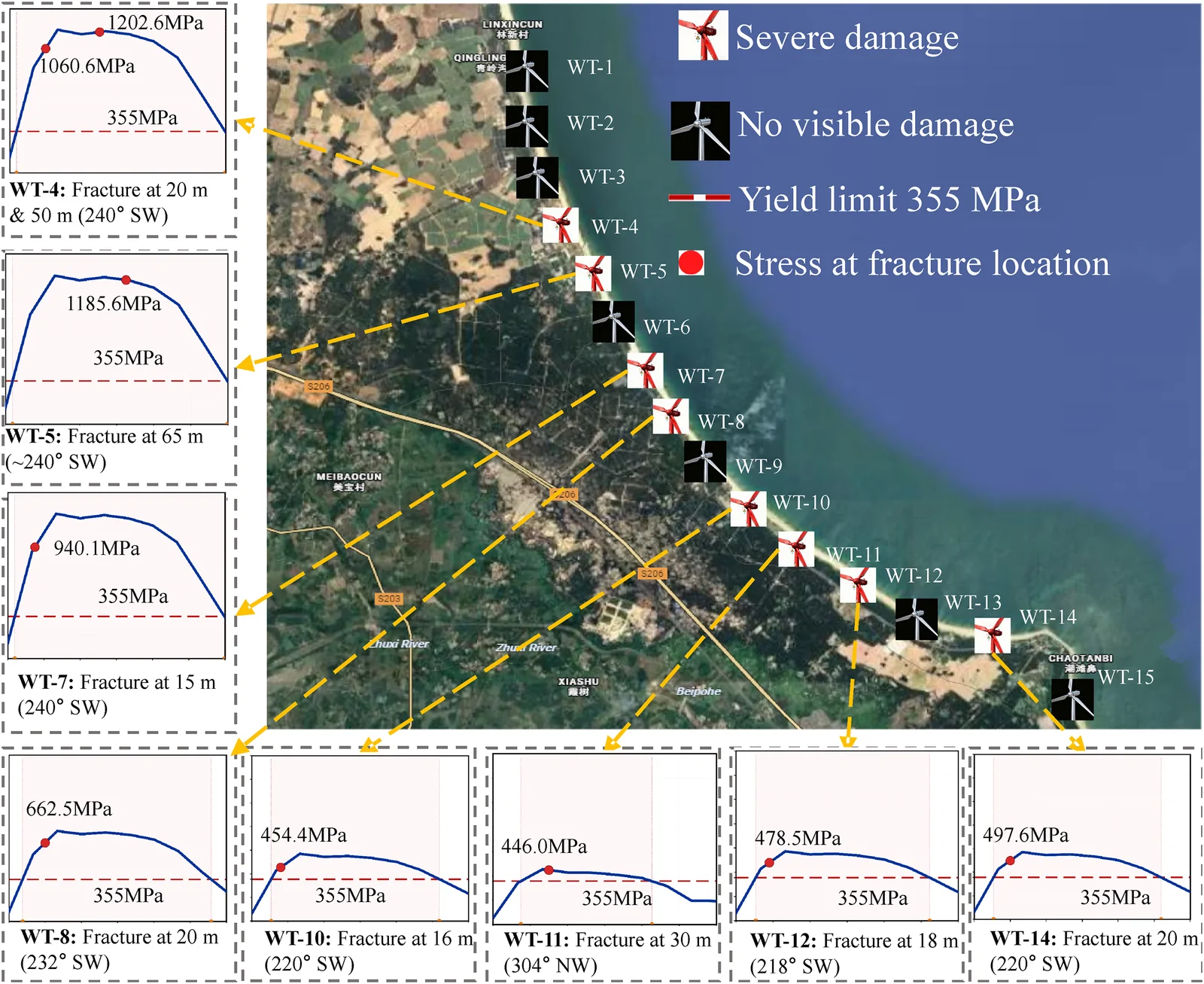
Zwind-computed linear-elastic tower stress profiles and their qualitative spatial comparison with image-estimated fracture locations under the reconstructed typhoon wind field Red dots indicate the calculated linear-elastic stresses at the approximate fracture heights identified from post-event images. The red dashed line denotes the 355 MPa yield-strength threshold of S355 steel, and values above this threshold are treated as indicators of yield-exceedance regions in the elastic stress-demand assessment.

Figure 5 therefore provides a qualitative link between the image-based damage assessment and the physics-based simulation analysis. To establish this link, the Reflector maps the approximate fracture heights identified in Figure 4 onto the Zwind-computed linear-elastic stress profiles in Figure 5. For all eight damaged-turbine cases included in the Typhoon Yagi analysis, the calculated stresses at the image-estimated fracture heights exceed the 355 MPa yield threshold. The image-estimated fracture heights and the Zwind-identified yield-exceedance regions therefore show qualitative spatial co-location along the tower height under the investigated typhoon conditions. Within the workflow, the Iwind agent organizes this simulation-observation comparison and summarizes the resulting correspondence. Previous studies have also shown that foundation response, including foundation uplift, can substantially influence wind-turbine tower demand under seismic loading.^32^

### Failure patterns by typhoon and earthquake

The Iwind agent employs the MCP to manage web crawling tools. This system collects publicly available reports from scientific literature, government databases, and official repositories, gathering both textual and image data on wind turbine damage caused by typhoons and earthquakes. The images are analyzed using a YOLO-based detector to identify and classify visible surface defects. At the case level, the typhoon-related damage is further grouped, based on the combined image and textual evidence, into three structural failure modes: blade fracture, tower buckling, and turbine overturning. The categorized damage data are combined with the corresponding textual information to generate structured summaries of the distinct failure modes observed.

As shown in Figure 6, subfigure (A) depicts damage caused by typhoons, while subfigure (B) shows the effects of earthquakes on wind turbines. The collected typhoon cases show blade fractures, tower buckling, and turbine overturning. Previous studies have identified control failure as one of several contributing factors in reported typhoon-related turbine failures.^5^^,^^6^ Furthermore, it is observed that these failure patterns exhibit distinct variations depending on the structural type of the wind turbine and its underlying foundation. The images highlight severe blade fractures and turbine overturning, illustrating the major failure modes observed during these disasters.

**Figure 6:**
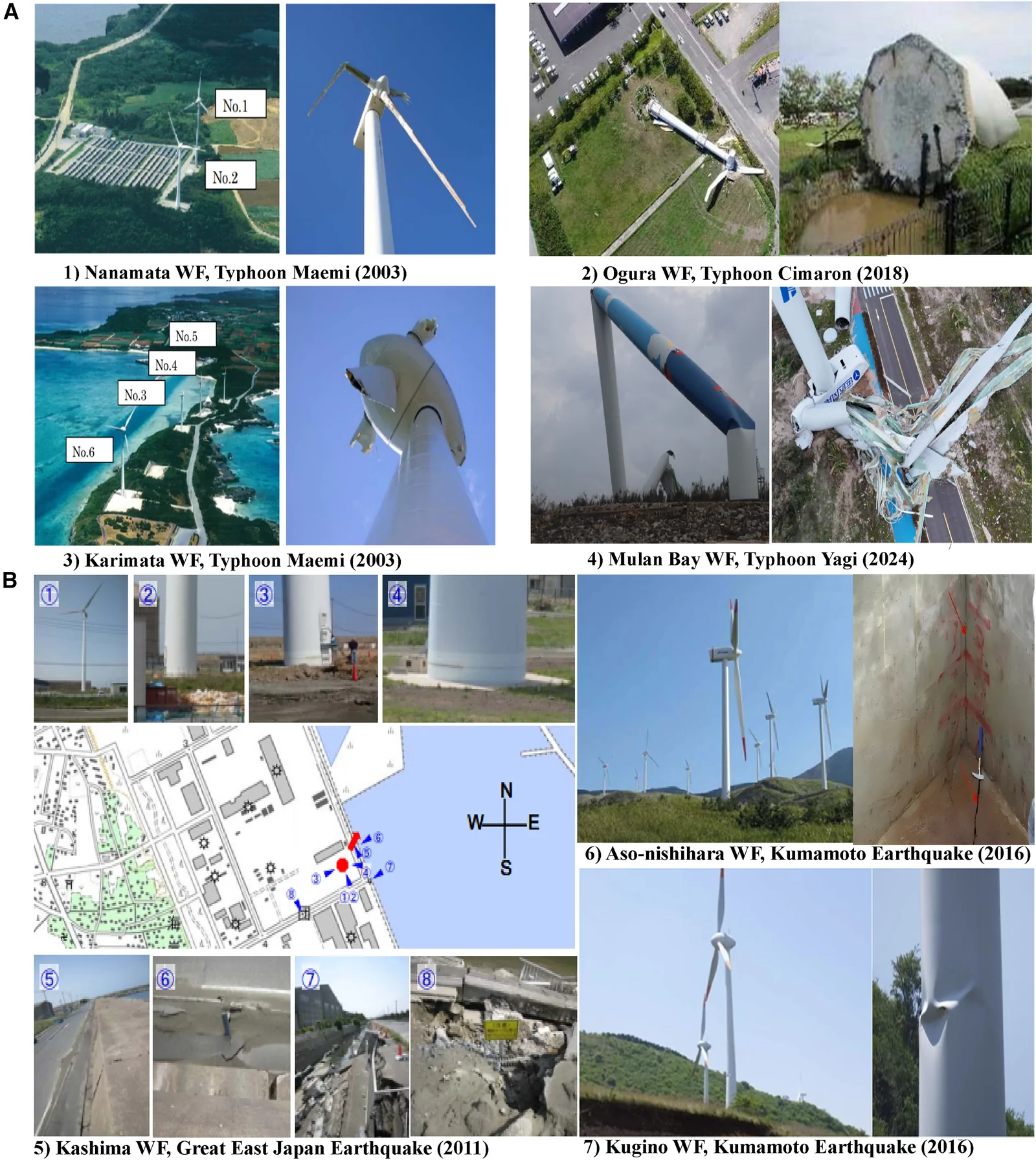
Wind turbine structural damage examples induced by typhoons and earthquakes (A) Typhoon-induced damage (section 1–4). (B) Earthquake-induced damage (section 5–7).

In contrast, the cases shown in Figure 6B, together with the prior study,^33^ indicate that some earthquake-damaged turbines exhibited more pronounced damage in the foundation or lower supporting structure, while the upper tower remained comparatively less damaged. This case-specific tendency highlights the importance of examining foundation and soil-structure response in post-event assessment and seismic design.

### Offline yaw-pitch response manifolds under typhoon and earthquake loading

Typhoons impose distributed aerodynamic loads on the rotor-tower system, whereas earthquakes introduce inertial excitation through the foundation. To examine the influence of parked-state configurations under each hazard, independent simulations were conducted for a prescribed set of fixed yaw-pitch combinations. Under the angle convention adopted in Zwind, the collective pitch angle was varied from 0° to 90° in 15° increments, covering the evaluated range from the unfeathered to the fully feathered state. Twelve yaw settings, ranging from −150° to 180° in 30° increments, sampled one complete 360° orientation cycle. Previous studies have shown that blade pitch and rotor yaw can substantially affect the structural and aeroelastic responses of parked offshore wind turbines under hurricane or extreme-wind conditions.^34^^,^^35^^,^^36^ Accordingly, the resulting grid of seven pitch settings and twelve yaw settings was adopted as a systematic discrete parameter sweep that balances angular coverage and computational cost. It was not intended to represent a standardized angular discretization or a continuous control optimization.

Both angles remained fixed throughout each simulated hazard process. For every yaw setting, the pitch state producing the lowest maximum structural stress among the seven evaluated fixed-pitch cases was selected. Connecting these discrete minima across the twelve yaw settings produced the offline, simulation-derived W- and M-shaped response manifolds. The selected states therefore represent minima within the prescribed discrete grid rather than global optima over a continuous yaw-pitch space. These manifolds characterize the structural responses associated with the evaluated parked states and support post-event state interpretation and hazard-region identification. They may also provide candidate references for pre-storm parked-state planning and future turbine-specific hazard-mitigation design. Practical implementation of these offline yaw-pitch state maps would require dedicated backup power, operable yaw and pitch actuators, adequate actuation rates, and turbine-specific safety verification.

### W-shaped yaw-pitch response manifold under typhoon loading

Current typhoon-resistant practice generally assumes that the yaw and pitch systems remain available during shutdown, allowing the turbine to maintain yaw = 0° and pitch = 90°. During extreme typhoons, however, grid loss or yaw-system damage may leave the turbine parked with a large yaw misalignment. We therefore evaluated a series of prescribed off-design states spanning the investigated yaw-pitch domain. Zwind calculated the structural response for each fixed yaw-pitch combination, and deterministic post-processing compared these cases to construct the W-shaped response manifold shown in Figure 7. For visualization in Figures 7 and 9, the color scale is limited to the S355 steel yield strength of 355 MPa. Simulated values above this threshold are mapped to the upper color limit to identify yield-exceedance states, while the original numerical values are retained. Under the investigated extreme wind speeds, aerodynamic thrust governs the tower-base bending moment. The resulting manifold therefore shows how the low-response pitch settings vary with yaw misalignment.

**Figure 7:**
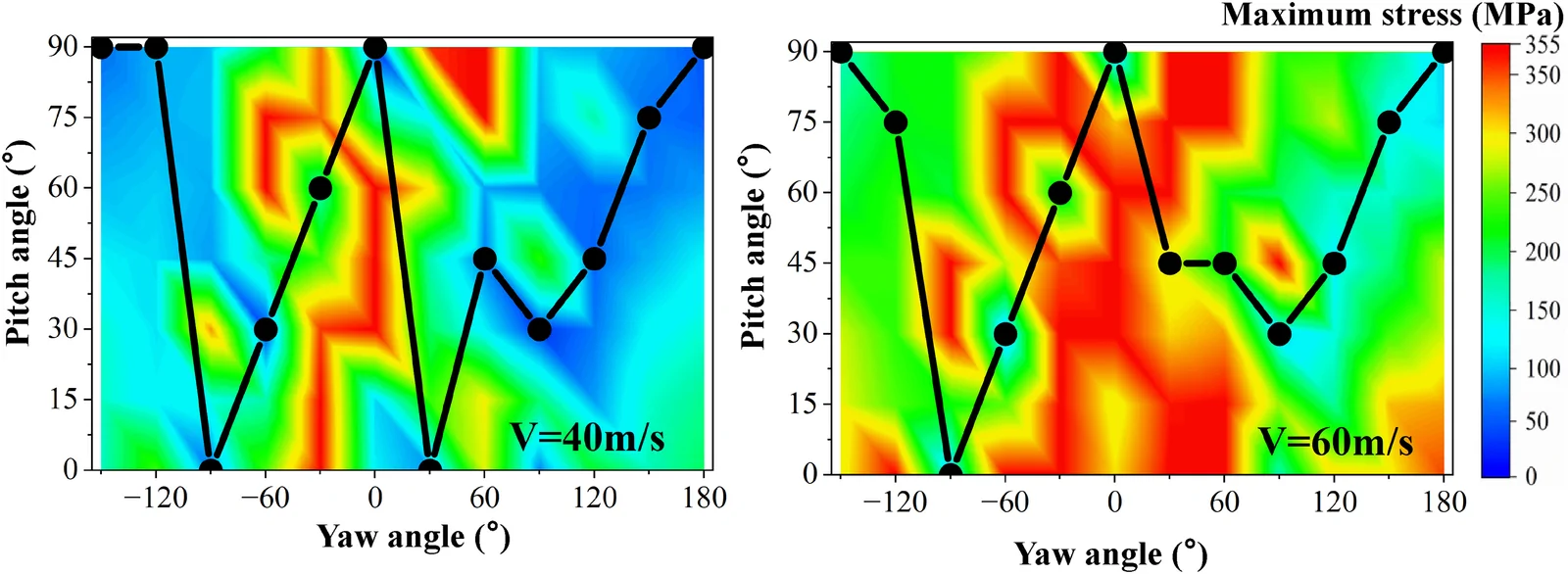
W-shaped yaw-pitch response manifold for selected parked states under extreme typhoon excitation The left and right images correspond to V = 40 and 60 m/s, respectively. The horizontal yaw and vertical pitch axes are in degrees (°), and the color field shows the simulated maximum stress in MPa. Solid dots mark fixed yaw-pitch combinations selected by cross-case response comparison; black lines connect these states to illustrate the geometric trend of the response manifold.

In the headwind and tailwind sectors (yaw = 0°, ±180°), the pitch-feathered state (pitch = 90°) produces lower simulated responses under the investigated conditions. Across the crosswind sectors (yaw = ±90°), the selected pitch settings shift progressively toward 0°–30°. Taken together, these cases reveal a direction-dependent response pattern associated with blade orientation under lateral inflow.

Furthermore, the W-shaped manifold is directionally asymmetric: the selected pitch angle at yaw = +90° differs from that at yaw = −90°. As shown in Figure 8, the IEA 10-MW blade exhibits spanwise variations in airfoil section, twist, and chord. Additional geometric sensitivity analysis described in the STAR Methods indicated that the projected blade area is sensitive to the spanwise twist distribution, while opposite yaw directions showed negligible projected-area differences. For the evaluated typhoon yaw-pitch combinations, the Zwind-computed response manifold retains the directional difference between yaw = +90° and yaw = −90° under the same structural model and environmental loading conditions.

**Figure 8:**
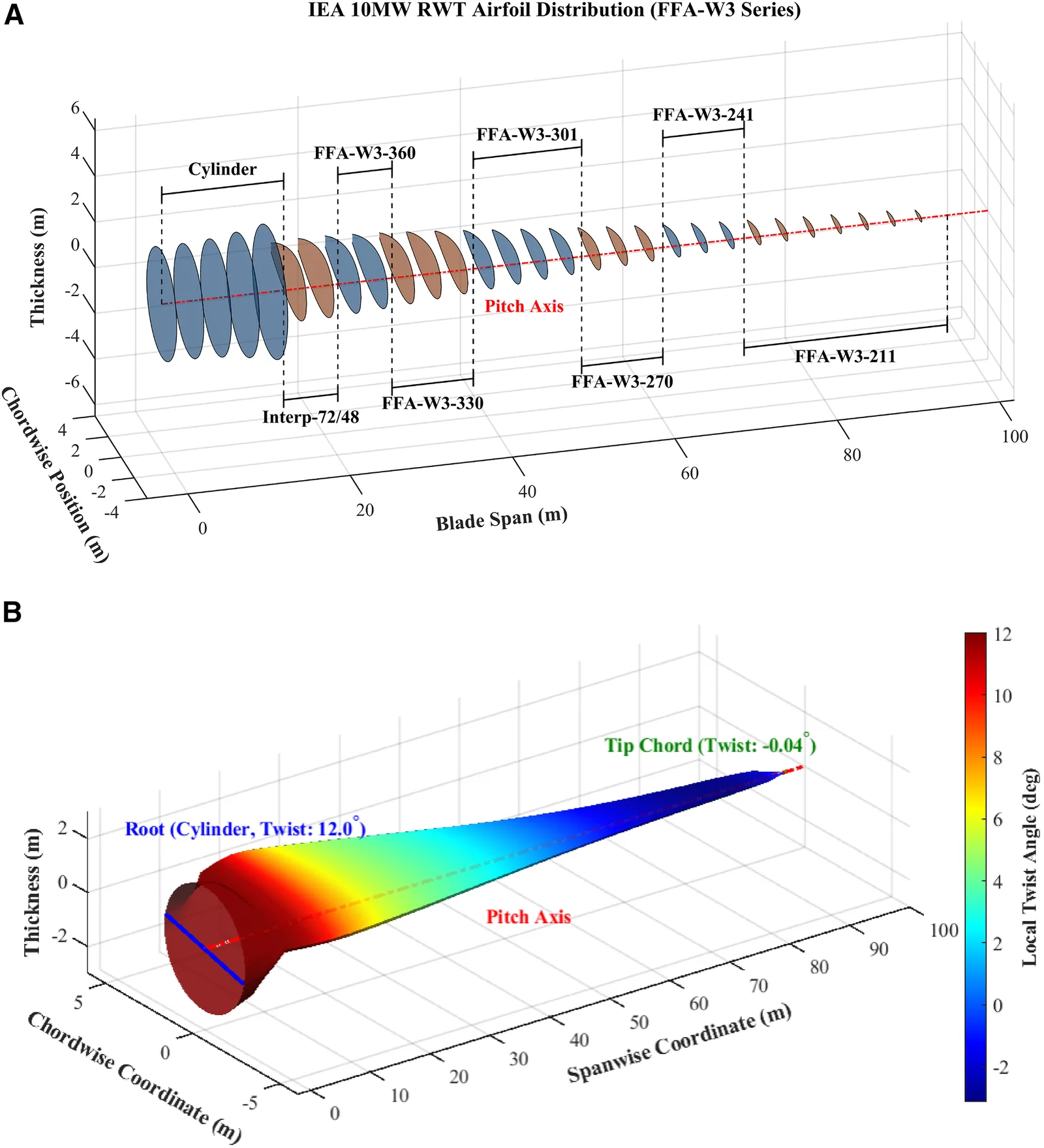
Spanwise blade geometry of the IEA 10 MW reference turbine (A) Discrete aerodynamic cross-sections based on the FFA-W3 airfoil series, showing the thickness transition from root to tip; (B) continuous three-dimensional blade envelope showing the spanwise variations in twist and chord.

### M-shaped yaw-pitch response manifold under earthquake loading

Typhoons load the structure from above, whereas earthquakes excite it from the base, leading to a different balance between aerodynamic damping and thrust. For the earthquake cases considered here, comparison of the fixed yaw-pitch states produces the nonlinear M-shaped structural-response manifold in Figure 9, which is a mirror image of the typhoon-induced W-shaped manifold. In this context, the mirror-image relation describes the reversed geometric trend traced by the selected yaw-pitch states across the yawed domain. For wind turbine structures subjected to prescribed PGAs of 1g and 2g at V = 3 m/s, seismic excitation dominates because aerodynamic loading is limited. The PGA = 2g case was prescribed in this study as a hypothetical extreme input for comparative structural-response analysis. Previous wind-turbine studies have likewise scaled ground motions to PGA levels of 1g, 2g, and 3g to investigate nonlinear structural responses and failure modes.^37^

**Figure 9:**
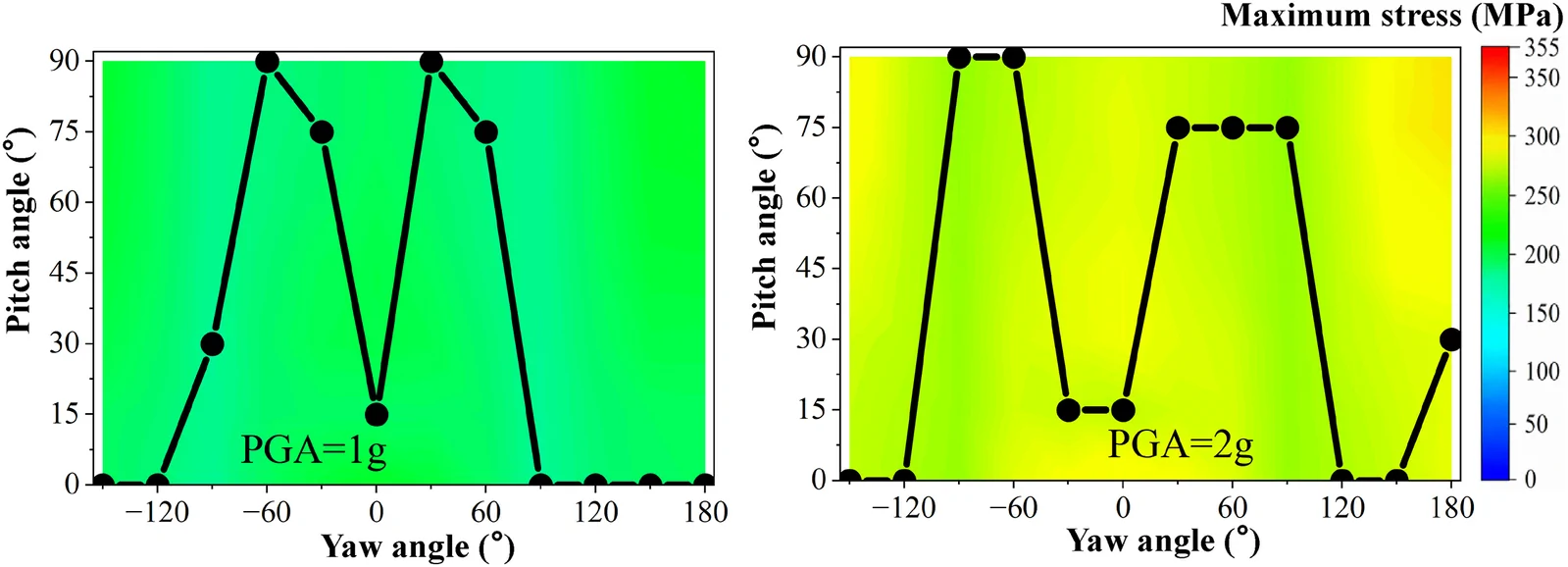
M-shaped structural-response manifold for selected parked states under severe seismic excitation The response manifold is evaluated at the prescribed low wind speed of V = 3 m/s. The left and right panels correspond to prescribed PGAs of 1g and 2g, respectively; the 2g case is treated as a hypothetical extreme scenario rather than a site-recorded intensity. The horizontal yaw and vertical pitch axes are in degrees (°), and the color field shows the simulated maximum stress in MPa. Solid dots mark fixed yaw-pitch combinations selected by cross-case response comparison; black lines connect these states to illustrate the geometric trend of the response manifold.

Under these conditions, the high-inertia rotor-nacelle assembly behaves as an inverted-pendulum system and amplifies fore-aft and lateral tower motion. Zwind computed the response of each fixed yaw-pitch case, and deterministic post-processing assembled the M-shaped manifold from the cross-case comparison. The Iwind-assisted workflow then organized these structured outputs and linked them to the yaw-dependent response pattern discussed below.

When yaw misalignment is close to 0° or ±180°, the cases with pitch angles near 15° exhibit lower structural responses. The associated response pattern is consistent with a greater aerodynamic-damping contribution during predominantly longitudinal tower motion. As yaw moves into the 30°–60° and −60° to −30° sectors, the low-response states shift toward pitch angles of 75°–90°, coinciding with a greater lateral-motion component.

Across the evaluated yaw-pitch combinations and seismic intensities, the structural response remains sensitive to the prescribed parked state. The M-shaped relation is therefore specific to the modeled turbine, loading conditions, and parameter ranges. As an offline state map, it provides a basis for parked-state assessment, hazard-region identification, and future turbine-specific seismic design.

The W- and M-shaped yaw-pitch relationships identified in this study should be regarded as simulation-derived candidate solutions within the evaluated turbine model, load cases, parameter ranges, and numerical constraints. They should not be interpreted as universally optimal or directly transferable industrial control strategies. Engineering application requires additional validation for specific turbine designs, site-specific wind and seismic conditions, control-system performance and availability, actuator constraints, and turbine-level safety requirements.

## Discussion

This study developed Iwind, a physics-simulation-oriented LLM-agent framework that transforms natural-language engineering requests into executable, traceable, and reproducible Zwind workflows. Rather than replacing the physical solver, the framework assigns the Iwind agent responsibility for intent recognition, domain-knowledge retrieval, task decomposition, parameter preparation, MCP-based tool invocation, deterministic post-processing, and execution feedback, while Zwind remains responsible for the underlying physics-based calculations. The central methodological contribution therefore lies in establishing a workflow-level connection between language understanding and engineering simulation, enabling an end-to-end process from user intent and solver-ready input generation to numerical execution, structured result extraction, and engineering reporting. On the Chinese WindBench benchmark, the full SFT + RL + RAG configuration outperformed the parent model across all reported metrics. Workflow-level evaluations further demonstrated the feasibility of automated simulation-input generation and end-to-end task completion, while the reported cases showed how image-derived damage observations could be organized alongside Zwind-computed responses for qualitative engineering interpretation. The publicly available code and reproducibility materials additionally allow the core natural-language-driven simulation workflow to be independently executed and examined.

The collected cases showed distinct damage patterns under typhoon and earthquake loading. Typhoons were associated with blade fractures, tower buckling, and turbine overturning, whereas some earthquake-damaged turbines exhibited more pronounced damage in the foundation or lower supporting structure. Parametric Zwind simulations further yielded nonlinear W- and M-shaped offline yaw-pitch response manifolds for the investigated typhoon and earthquake cases, respectively. These results support comparison among fixed parked states and identification of high-response regions under the evaluated conditions.

The Iwind agent should therefore be regarded as a near-application research framework rather than a fully deployed industrial system. The GitHub repository identified in the Code availability section provides the agent code, configuration files, evaluation scripts, and representative input-output examples. The released case materials also include a compiled Zwind executable and the corresponding input files to support case-level reproduction. The Zwind source code remains subject to commercial-confidentiality and software-licensing restrictions. Transfer of the agent-side workflow to another numerical solver requires a solver-specific MCP interface, compatible input-output adapters, and independent verification of numerical equivalence.

In extreme-disaster applications, qualified experts must verify the input conditions and simulation-model selection, screen abnormal outputs, confirm image-based damage findings, approve control recommendations, and complete safety validation before implementation. The current framework supports assisted engineering analysis but does not replace expert judgment or established safety-review procedures.

The W- and M-shaped patterns should be interpreted as simulation-derived offline response manifolds assembled from independent cases with fixed yaw and pitch angles, rather than as trajectories generated by an implemented real-time controller. Their engineering application requires systematic evaluation of sensitivity to wind speed, turbulence intensity, ground-motion characteristics, and turbine parameters; comparison with conventional shutdown strategies; independent-solver cross-validation; and assessment of blade-root loads, actuator rate limits, pitch-system demand, control-system availability, and multi-objective trade-offs between tower safety and subsystem loading.

Future studies will further investigate these aspects to improve the engineering applicability of the proposed framework. Broader sensitivity analyses incorporating additional wind conditions, turbulence characteristics, ground-motion records, and turbine parameters will help evaluate the robustness and transferability of the identified response patterns. Comparative investigations between the offline yaw-pitch response manifolds and conventional shutdown strategies, together with cross-validation using independent simulation tools when appropriate, will provide further insight into their engineering interpretation. In addition, integrating actuator constraints, structural safety indicators, subsystem loading characteristics, and control-system requirements into a unified multi-objective trade-off analysis framework will facilitate the extension of Iwind toward engineering decision support applications.

The typhoon and earthquake simulations in this study were designed as separate hazard scenarios. The earthquake cases prescribed a low wind speed of V = 3 m/s, whereas the typhoon cases considered wind speeds of V = 40–60 m/s. This configuration enabled separate examination of the structural responses associated with the two hazard types under the investigated conditions. Building on the directional wind-seismic interaction reported by Ishihara et al.,^14^ future work will extend the framework to simultaneous strong-wind and earthquake loading by incorporating site-specific wind fields, ground motions, directional combinations, and turbine operating states.

Zwind includes hydrodynamic-load modeling, which provides a basis for extending the present framework to offshore wind-turbine damage cases. Such validation requires a documented event with sufficiently complete field records. Previous numerical studies examined the responses of floating and monopile offshore wind turbines under the environmental conditions of Typhoon Mangkhut and Super Typhoon Chan-hom, respectively.^38^^,^^39^ Wind Power Kamisu Phase 1 was also exposed to the 2011 Great East Japan Earthquake and an approximately 5 m tsunami, after which inspections reported no system damage before operation resumed.^40^ These studies and records demonstrate the relevance of typhoon, earthquake, and tsunami hazards to offshore wind turbines. However, the currently available information does not support a like-for-like reconstruction that jointly specifies the loading histories, operating conditions, turbine and foundation parameters, and measured post-event damage. The present study therefore provides a scenario-based methodological assessment. Future work will prioritize field validation against a documented offshore damage or failure event when the required records become available.

It is worth noting that wind turbine research is undergoing a paradigm shift from disaster analysis to full life cycle O&M. In the future, our focus will expand from failure analysis to sustainable empowerment. In response to the challenges of poor accessibility and extremely high O&M costs of offshore wind turbines, traditional post-disaster repair models are no longer sustainable. This agent must continuously evolve to possess predictive capabilities, not only to analyze how structures fail but also to predict when and where damage may occur, and to optimize decision-making to achieve the safest operation at the lowest cost, thereby pioneering a new path for intelligent O&M of deep-sea wind power.

### Limitations of the study

In this study, the Iwind agent is physics-informed through its workflow-level integration with physics-based numerical simulation, solver-compatible parameter definitions, predefined engineering constraints, deterministic post-processing procedures, and simulation-observation comparison. The Iwind agent interprets engineering requests, organizes input parameters, invokes connected tools, and summarizes the returned evidence. Zwind calculates the corresponding loads and structural responses, while the visual module extracts observable damage features from post-event images. Domain-specific model adaptation and RAG support task interpretation and evidence retrieval, whereas the physical quantities reported in this study are obtained from numerical simulation and predefined deterministic analysis procedures.

The physical analyses are conducted using the selected turbine models, reconstructed or prescribed hazard inputs, and the investigated parameter ranges. Typhoon and earthquake loading are considered as separate hazard scenarios. For the tower assessment in typhoon damage assessment, Zwind uses linear-elastic beam elements to calculate global structural responses and sectional elastic stress demand along the tower height. The yield strength of S355 steel, 355 MPa, is used to identify yield-exceedance regions, and stresses above this threshold are interpreted as elastic demand indicators for locating high-demand sections. This representation supports global response assessment and critical-height identification, while detailed local shell deformation and post-yield stress redistribution require a higher-fidelity nonlinear structural model.

Post-event fracture heights and visible C- or Z-shaped damage morphologies are estimated by the visual module from collected images and are treated as approximate observational evidence. Their interpretation depends on image resolution, viewing angle, occlusion, and the geometric references available in each image. Automated execution further assumes that the required inputs are complete, physically reasonable, and compatible with the connected MCP tools and solver interfaces. The Iwind agent performs parameter checks and organizes traceable execution records under these conditions, while qualified engineering review remains necessary for abnormal outputs and safety-related interpretation.

These assumptions define the scope of the reported findings. The comparison in Figure 5 supports a qualitative spatial correspondence between image-estimated fracture heights and Zwind-identified yield-exceedance regions. The W- and M-shaped yaw-pitch manifolds are assembled from independent simulations in which the yaw and pitch settings remain fixed within each case; they therefore support offline comparison of parked states and identification of high-response regions under the investigated typhoon and earthquake conditions. The reported agent performance applies to the tested task categories, connected tools, and predefined solver interfaces. Extension to other turbine configurations, sites, combined hazards, or operational control systems requires corresponding physical inputs, interface adaptation, and case-specific validation.

Additional methodological details are provided in Figures 10, 11, 12, 13, and 14. Figure 10 illustrates the thought–action–observation workflow of the Iwind agent, showing how user requests are converted into executable tasks, connected tools are invoked through MCP, and returned results feed back into the workflow. Figure 11 summarizes the domain-specific model development, evaluation, and integration workflow, including data curation and WindBench construction, domain pre-training, supervised fine-tuning, response-quality grading, reward-guided reinforcement learning, and the genre–audience data-construction strategy. Figure 12 shows the distribution of the 15 task categories and their sample counts in the 15,000-pair WindBench benchmark. Figure 13 presents the data-curation and multi-path RAG retrieval architecture, including parallel retrieval, fusion, and re-ranking. Figure 14 depicts the Zwind software architecture and numerical solution process, from input and external-load evaluation through Kane-based equation solution and time integration to response output. The corresponding implementation details are described in STAR Methods.

**Figure 10:**
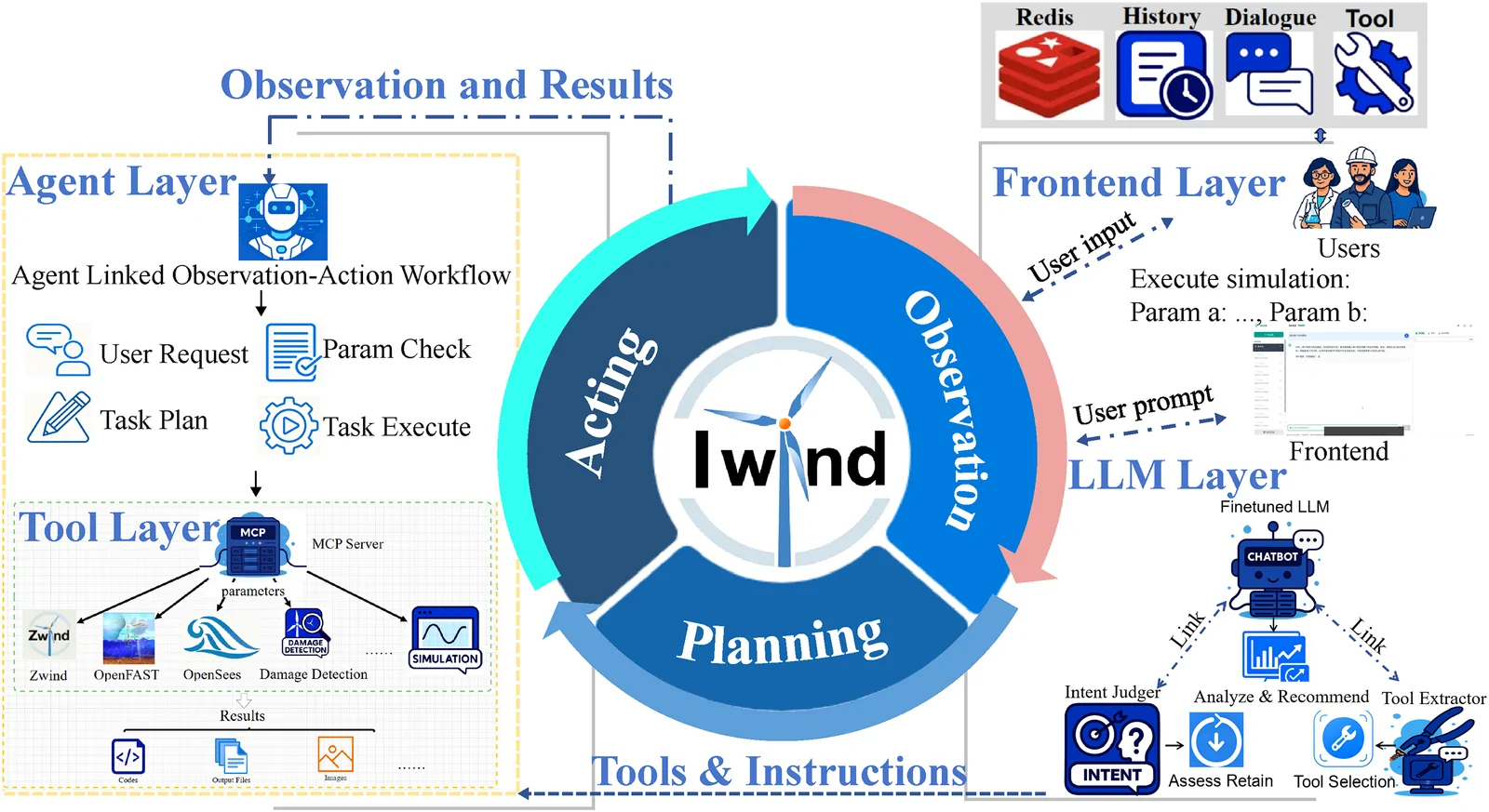
Thought-action-observation workflow of the Iwind agent The frontend layer receives user requests, the LLM and agent layers interpret and convert them into executable tasks, and the tool layer invokes Zwind and other connected tools through MCP. Returned results re-enter the planning-action-observation loop for verification and reporting. The Iwind agent performs interpretation and orchestration, whereas the connected tools perform the corresponding numerical or perception tasks.

**Figure 11:**
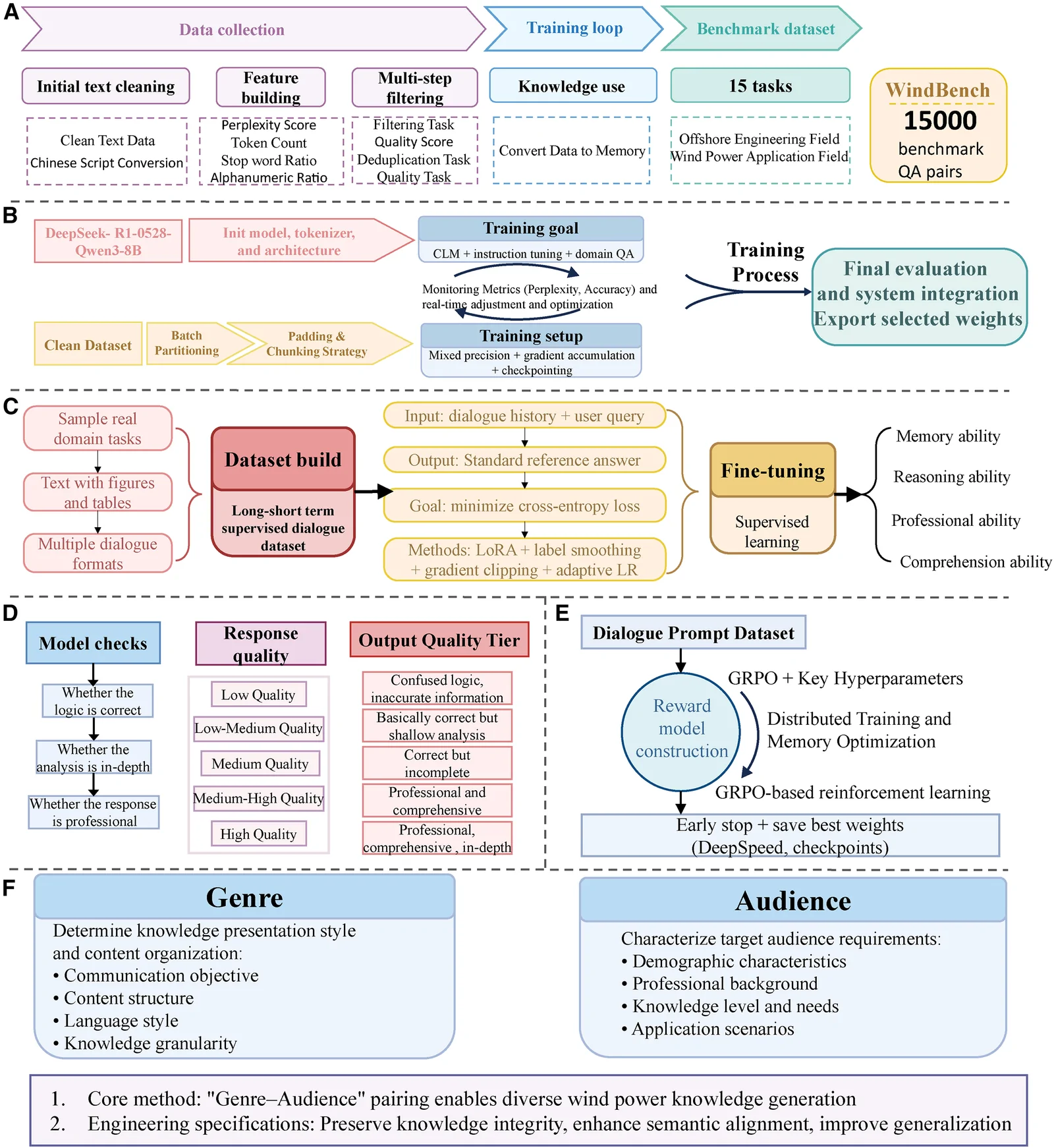
Domain-specific model development, evaluation, and integration workflow for the Iwind agent (A–F) summarize data curation and WindBench construction, domain pre-training, supervised fine-tuning, response-quality grading, reward-guided reinforcement learning, and the genre-audience data-construction strategy, respectively. The cleaned domain corpus provides the input for domain pre-training, domain-task dialogs support supervised fine-tuning, and quality-annotated response groups provide the supervision for reward-model construction. The resulting reward signals guide GRPO-based reinforcement learning and model selection. The candidate configurations are evaluated on WindBench, an independent Chinese benchmark containing 15,000 question-answer pairs across 15 task categories, and the selected model is integrated with RAG-assisted retrieval and MCP-based tool orchestration.

**Figure 12:**
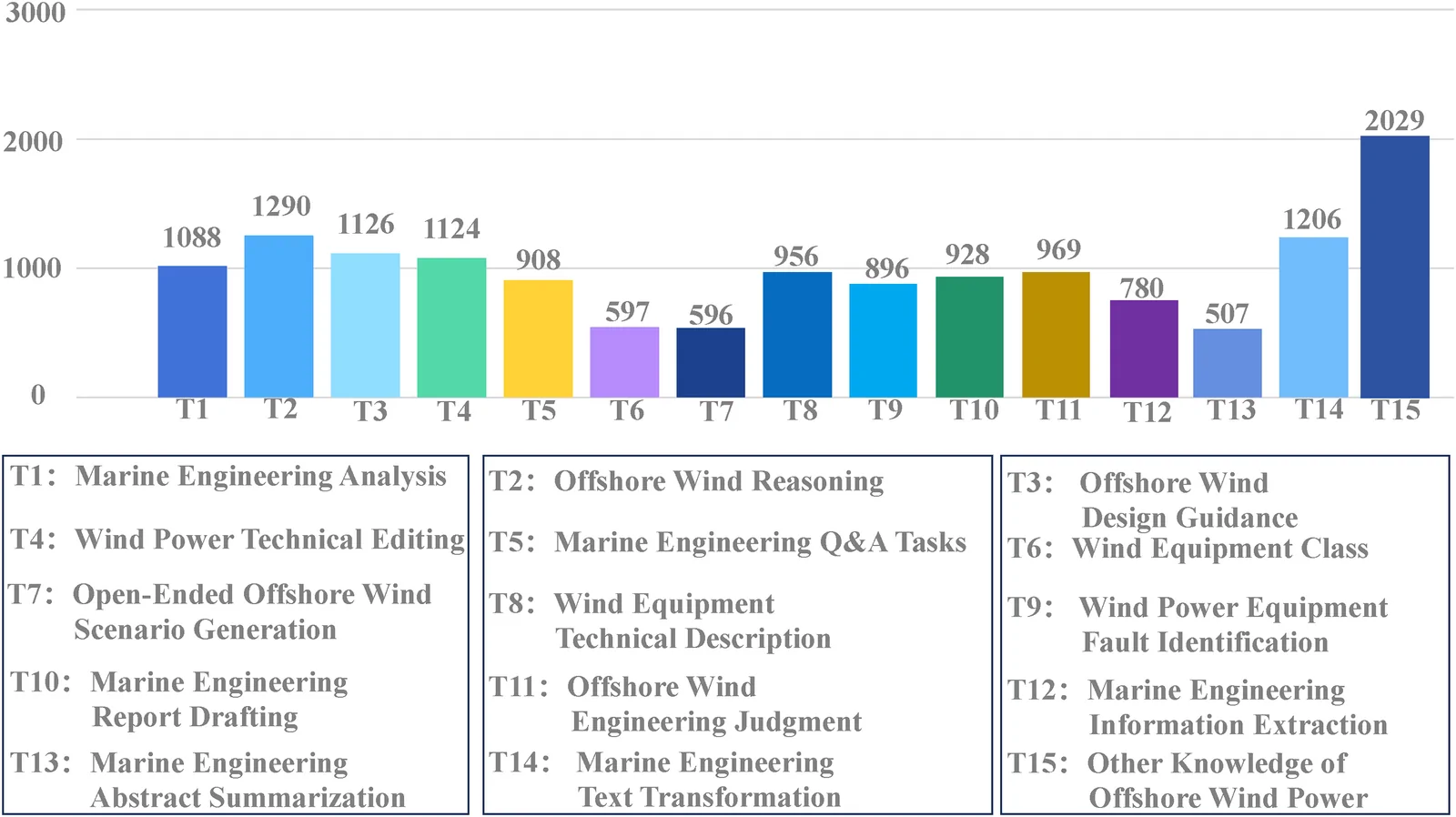
Task distribution of the WindBench benchmark dataset The figure shows the 15 WindBench task categories and their corresponding sample counts within the 15,000-pair benchmark.

**Figure 13:**
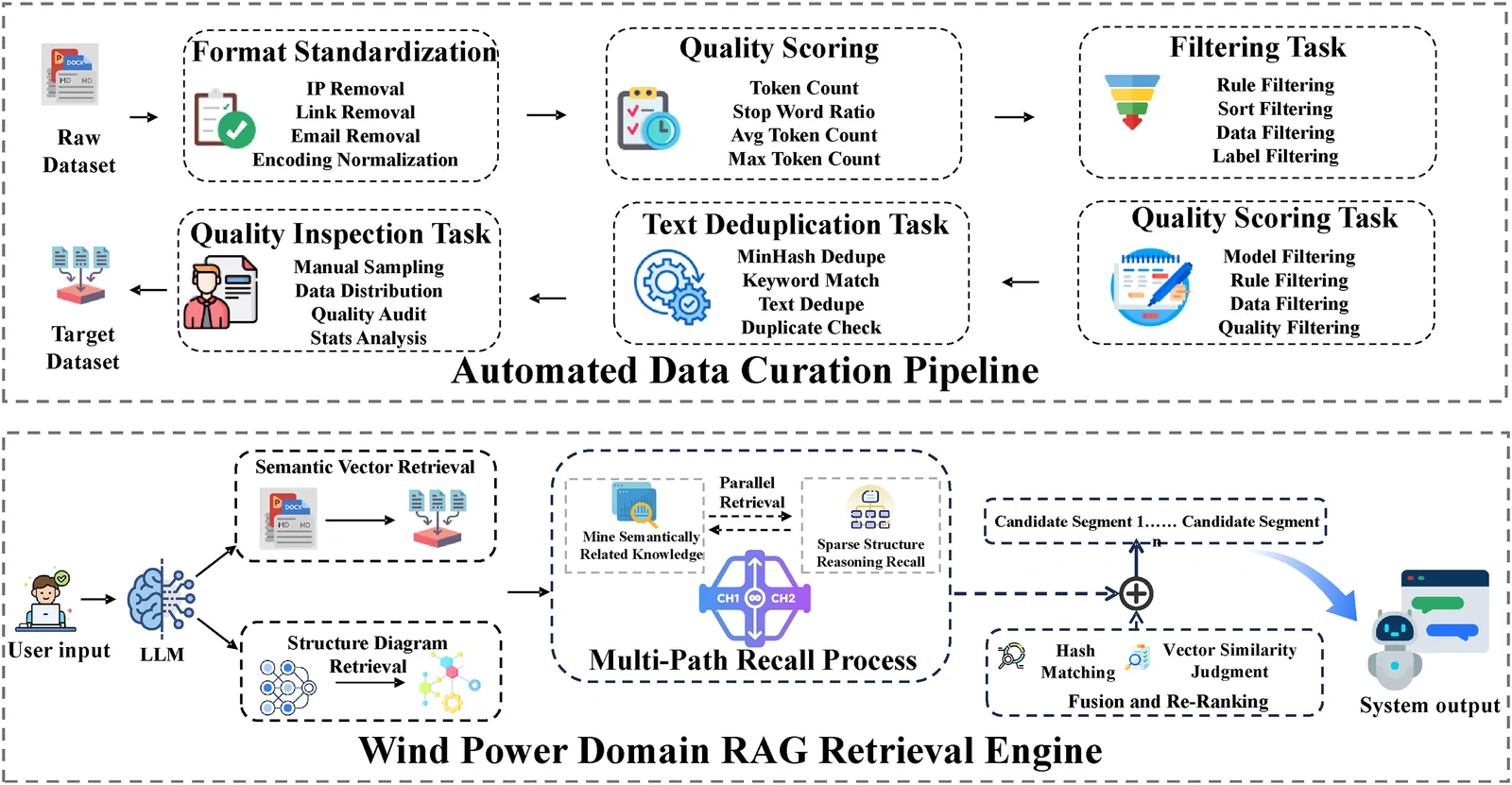
Data-curation and retrieval architecture of the Iwind agent The upper part summarizes the automated data-curation process, while the lower part shows the multi-path RAG retrieval process, including parallel retrieval, fusion, and re-ranking.

**Figure 14:**
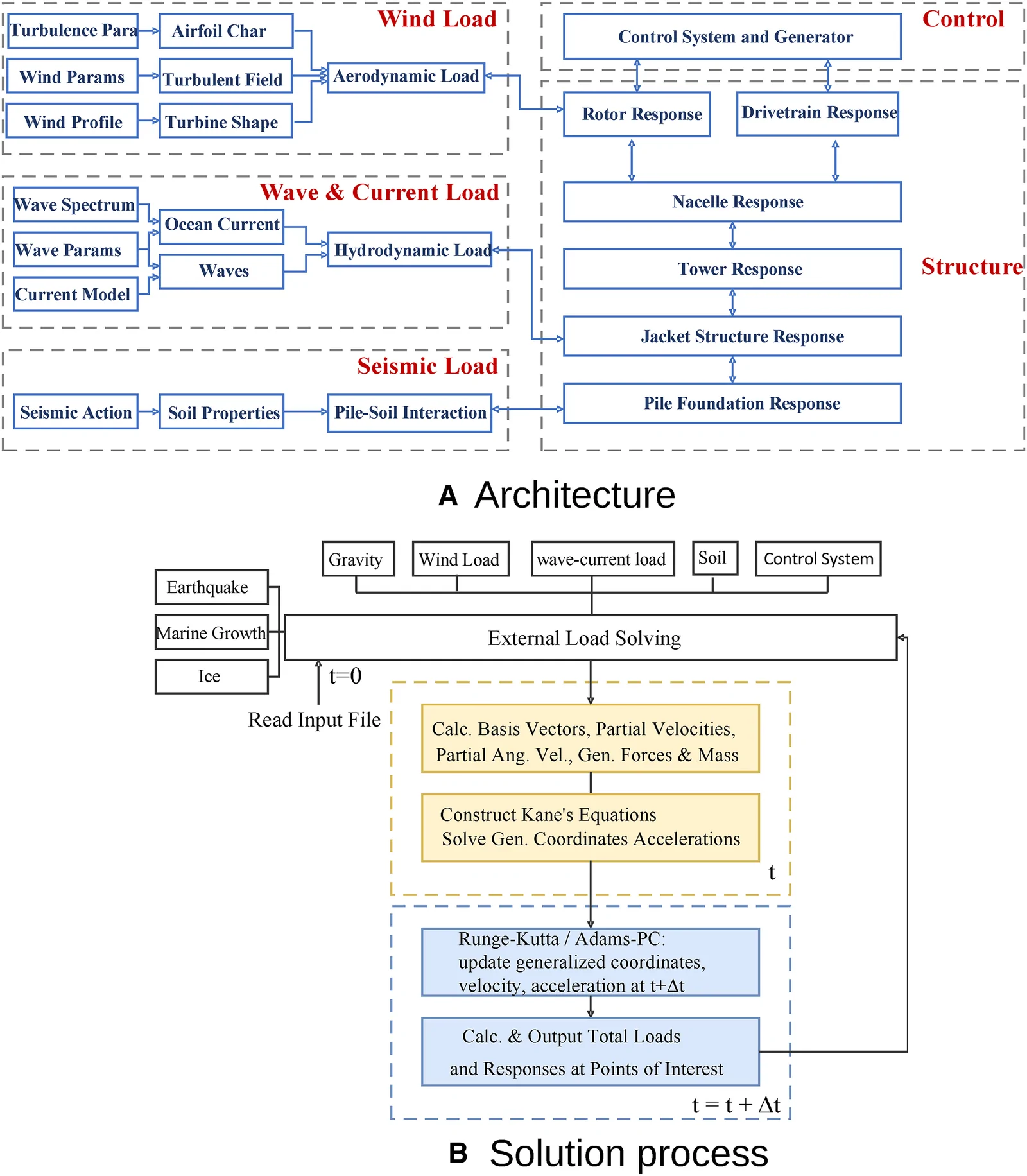
Software architecture and solution process of Zwind (A) Shows the software architecture of Zwind, and (B) shows the numerical solution process from input and external-load evaluation to Kane-based equation solution, time integration, and response output.

## Resource availability

### Lead contact

Further information and requests for resources should be directed to and will be fulfilled by the lead contact, Lilin Wang (lilin.wang@zju.edu.cn).

### Materials availability

This study did not generate new unique materials.

### Data and code availability

• The public code and reproducibility materials supporting the Iwind workflow are available at https://github.com/linjunfuzju/iwind.git. The repository includes the implementation of the Iwind agent workflow, configuration files, single-case and batch simulation scripts, deterministic post-processing and visualization tools, representative input-output examples, and detailed execution instructions. The reproduce/directory additionally provides a Docker archive containing the runnable Zwind executable and the required simulation environment. Together with the complete simulation dataset described below, these publicly available resources enable researchers to independently execute and verify the core workflow reported in this study, from natural-language task input and simulation configuration generation to Zwind execution, result output, and deterministic post-processing. Certain model-development materials, including domain-adapted model parameters and associated training resources, are not included in the current public release. These materials contain commercially sensitive and potentially protected technical content and remain subject to final intellectual-property evaluation, third-party licensing review, and legal compliance assessment. Their public distribution is therefore temporarily restricted. Following formal publication of the manuscript and completion of the relevant reviews, publicly releasable versions will be provided within the scope permitted by applicable intellectual-property, confidentiality, and licensing conditions.
• The simulation data generated in this study are publicly available through Science DataBank (https://doi.org/10.57760/sciencedb.45382). The deposited dataset contains the simulation files generated from parameterized Zwind analyses of an offshore wind turbine under prescribed extreme-loading scenarios, together with the associated simulation outputs and metadata for data verification.
• Any additional information required to reanalyze the data reported in this study is available from the lead contact upon request.

## Acknowledgments

This work was supported by the Self-funding of the State Key Laboratory of Ocean Sensing (“Precision Sensing Technologies and Systems for Unmanned Operation and Maintenance of Far-Offshore Wind Farms”; Project No. OSZ-2026ZX01), the Excellent Young Scientists Fund (Overseas), the 10.13039/501100001809National Natural Science Foundation of China (52409144, 52238008), the 10.13039/100022963Key R&D Program of Zhejiang Province of China (2025C01172), the Fujian Provincial Department of Science and Technology Major Special Project (grant no. 2025HZ024032), and the Major Science and Technology Project of Fuzhou (grant no. 2025-ZD-013).

## Author contributions

L.W. and L.W. conceived the study, designed the research framework, developed the methodology, revised the manuscript, and provided overall guidance and strong support for this study. L.F. and Z.X. contributed to large language model training and fine-tuning, the design and implementation of the agent architecture, data processing, visualization, experimental design, result interpretation, and preparation of the original manuscript draft. S.Z. contributed to data processing, visualization, interpretation of the results, and revised the manuscript. H.C. participated in the technical analysis and manuscript revision. All authors reviewed and approved the final manuscript.

## Declaration of interests

The authors declare the following patent interests related to the work reported in this manuscript.

Several authors are named inventors on the granted Chinese invention patent entitled “Application Method, Apparatus, Device, and Medium for Large Language Models in Offshore Wind Power and Ocean Engineering” (patent no. ZL 2026 1 0572389.X; application no. 202610572389.X; filed April 28, 2026; granted July 31, 2026). F.L. is the first inventor, and Zhejiang University is the patentee.

Chinese patent application no. 202610572421.4, filed April 28, 2026, entitled “Intelligent Simulation Method, Apparatus, Device, and Medium for Offshore Wind Power Operation and Maintenance,” received a “Notification of Grant of Invention Patent” from the China National Intellectual Property Administration dated August 11, 2026. The accompanying registration notice states that the patent right will be registered, certificated, and announced after the required registration procedure is completed.

One or more authors are named inventors on the following pending patent applications related to the work reported in this manuscript. All of these applications are assigned to Zhejiang University.

1. Chinese patent application no. 202610686858.0, filed May 18, 2026, entitled “Knowledge-Enhanced Retrieval-Driven Multimodal and Multi-Terminal Intelligent Question-Answering Method, Apparatus, Medium, and Product for Wind Power.”
2. Japanese patent application no. 2026–083020, filed May 18, 2026, entitled “Intelligent Simulation Method, Apparatus, Device, and Medium for Offshore Wind Power Operation and Maintenance.”
3. Chinese patent application no. 202610827048.2, filed June 9, 2026, entitled “Artificial-Intelligence-Based Method, Device, and Medium for Assessing Typhoon-Induced Disaster Risk and Resilience of Offshore Wind Turbines.”
4. Chinese patent application no. 202610829523.X, filed June 9, 2026, entitled “Method, System, Device, Medium, and Product for Constructing Large Language Model Training Datasets for the Wind Power Sector.”

The authors declare no other competing interests.

## STAR★Methods

### Key resources table

**Table:** 

REAGENT or RESOURCE	SOURCE	IDENTIFIER
**Deposited data**		

Iwind simulation dataset	This study	Science DataBank DOI: https://doi.org/10.57760/sciencedb.45382
WindBench benchmark dataset	This study	GitHub repository: https://github.com/linjunfuzju/iwind/tree/main/qa_data

**Software and algorithms**		

DeepSeek-R1-0528-Qwen3-8B	DeepSeek	https://huggingface.co/deepseek-ai/DeepSeek-R1-0528-Qwen3-8B
QRM-Llama3.1-8B-v2	Nicolai Dorka	https://huggingface.co/nicolinho/QRM-Llama3.1-8B-v2
BGE-M3	Beijing Academy of Artificial Intelligence	https://huggingface.co/BAAI/bge-m3
Qwen3-VL-8B-Instruct	Qwen	https://huggingface.co/Qwen/Qwen3-VL-8B-Instruct
Iwind agent workflow code	This study	GitHub repository: https://github.com/linjunfuzju/iwind

### Experimental model and study participant details

Omitted as our study does not involve biological models.

### Method details

#### Physics-informed integration of the iwind agent and Zwind

Figure 10 summarizes how the Iwind agent connects natural-language engineering requests with traceable tool-execution workflows. It also defines the functional boundary between agent-side orchestration and the numerical or perception tasks performed by the connected tools.

Natural-language requests and user-defined simulation parameters first enter the Frontend Layer, which also maintains the interaction history and tool metadata required throughout execution. Within the LLM Layer, user intent is identified, relevant context is preserved, candidate actions and tools are selected, and the arguments required for invocation are extracted. Parameter validity is then checked by the Agent Layer. Based on the interpreted request, this layer constructs an executable workflow, dispatches individual tasks, and uses each returned observation to determine the subsequent action. Tool execution is handled through MCP, which connects the framework to Zwind, OpenFAST, OpenSees, the damage-detection module, and other available tools. Status codes, output files, images, and numerical results are subsequently returned to the agent. Collectively, these operations establish a traceable planning–action–observation cycle.^41^ Within this division of responsibilities, the Iwind agent interprets tasks and orchestrates workflows, while Zwind and the connected tools perform the corresponding numerical simulation or perception tasks.

Three groups of enabling components were further adapted and coordinated to support this natural-language-driven engineering workflow. Domain-specific model adaptation and multi-path RAG provide engineering knowledge retrieval and convert natural-language requirements into executable tool-call plans. MCP, in turn, standardizes solver invocation, parameter transfer, input–output specifications, and error handling, thereby supporting traceable and recoverable execution. Visual evidence is processed by external modules, including a YOLO-based wind-turbine damage detector and Qwen3-VL-8B-Instruct, which transform images into structured observations that can be associated with the physical responses computed by Zwind. Accordingly, the principal methodological contribution of this study lies in the workflow-level coordination of language understanding, knowledge retrieval, task orchestration, visual perception, and physics-based simulation.

#### Domain-specific model training

This subsection presents a systematic domain-specific training framework for offshore wind power and marine engineering. The framework aims to strengthen the capabilities of Iwind agent in knowledge representation, task decomposition, and tool orchestration. Built on a general-purpose base model, the framework progressively incorporates domain-specific corpora, task-annotated datasets, and structured reward signals. This staged training yields a model that can generate professional responses under RAG evidence constraints and can reliably map natural language requirements into executable MCP call plans.

Figure 11 summarizes the sequence linking domain pre-training, supervised fine-tuning,^42^ reward-model construction,^43^ reinforcement learning,^44^ final evaluation, and system integration. It also distinguishes the resources used for model development from the independently constructed WindBench benchmark.

The model-development resources comprised a quality-controlled corpus derived from 12,000 screened source documents and containing approximately 180 million cleaned tokens, 50,000 domain-specific instruction–response pairs for supervised fine-tuning, and 12,000 quality-annotated response groups for reward-model training. WindBench was constructed independently as a held-out Chinese benchmark containing 15,000 expert-reviewed question–answer pairs across 15 task categories. Its source materials were separated from those used for the domain corpus, supervised fine-tuning, reward-model training, and the RAG knowledge base, thereby preventing direct overlap between model development and evaluation. The implementation details of the five stages are provided in the following subsections.

#### Benchmark dataset phase

This study systematically collected multi-source corpora for offshore wind power and marine engineering, as shown in Figure 12, including technical documents, operational logs, research articles, equipment manuals, and engineering reports. After denoising, segmentation, and format standardization, the raw data were further processed using rule-based and model-based filtering, keyword matching, and locality-sensitive deduplication to construct a quality-controlled target dataset.

WindBench was independently constructed as a held-out Chinese benchmark covering marine engineering and offshore wind power. One group of task categories addresses marine engineering applications, including design and analysis, construction and installation, operation, maintenance, and monitoring. The other group addresses offshore wind power tasks, including turbine reasoning, project design, technical documentation, component classification, and fault identification. WindBench contains 15,000 Chinese question–answer pairs across 15 task categories. The benchmark includes both quantifiable objective questions and open-ended technical questions, and each pair has one expert-reviewed reference answer.

The source documents used to construct WindBench were separated from those used for the domain corpus, supervised fine-tuning dataset, reward-model training dataset, and RAG knowledge base. Accordingly, none of the WindBench questions, reference answers, or source documents was used for model training or retrieval. The 15 task categories were defined by multiple domain experts according to recurring requirements in offshore wind power and marine engineering. The sample counts were not forced to be equal across categories; instead, their distribution was determined through expert discussion according to the relative importance of the tasks in the intended applications, as shown in Figure 12, which presents the 15 subtask categories and their corresponding sample counts. Each question–answer pair was manually reviewed and corrected by multiple domain experts before inclusion.

#### Domain pre-training phase

During domain pre-training, DeepSeek-R1-0528-Qwen3-8B^45^^,^^46^ served as the common parent model for all evaluated configurations. Its 8B parameter scale was selected to balance domain-adaptation capacity with the available local computing resources and prospective edge-deployment requirements. The quality-controlled domain corpus was derived from 12,000 screened source documents and contained approximately 180 million cleaned tokens. The corpus was subsequently segmented and encoded. The model was then trained with an autoregressive language modeling objective. This stage emphasized domain-specific terminology and cross-document logical relationships in engineering contexts. Multi-GPU parallelism and checkpoint resumption were used throughout training. The team continuously monitored perplexity and loss curves to support stable convergence and reduce overfitting risk.

#### Supervised fine-tuning phase

Throughout supervised fine-tuning, the model was further trained using 50,000 domain-specific instruction–response pairs covering typical engineering tasks, including single-turn instruction following, short-turn questioning, and cross-component reasoning. The training objective was to minimize cross-entropy loss. A parameter-efficient low-rank adaptation (LoRA)^47^ strategy and gradient accumulation were used to improve optimization stability. Validation combined automated metrics and expert evaluations to assess answer consistency, technical expression accuracy. This phase yielded a model that performed well on domain-specific dialogue and reasoning tasks.

#### Reward model construction phase

During reward-model construction, 12,000 quality-annotated response groups were used to train the quality-scoring model. Response quality was categorized into five levels according to professional accuracy, logical rigor, and linguistic standardization. The reward-model backbone was derived from the Llama model family^48^ and the Llama 3.1 architecture.^49^ The quality-scoring model used in this study was initialized from the publicly available nicolinho/QRM-Llama3.1-8B-v2 checkpoint^50^ and was subsequently trained to distinguish among five response-quality levels.

#### Reinforcement learning phase

During the reinforcement learning phase, the supervised fine-tuned model served as the initial policy and generated multiple candidate responses from prompt data without reference answers. Each candidate response was independently evaluated by the reward model, and the aggregated scores were then used to guide policy optimization. Policy optimization was performed using the GRPO method,^51^ which introduces truncated probability ratios and an entropy regularization term to balance optimization strength and output diversity. Distributed acceleration and early stopping mechanisms were applied to maintain stable validation performance while preserving the optimal model weights. This closed-loop optimization process improves generation quality and enhances robustness in engineering question-answering scenarios.

#### Final evaluation and integration phase

In the final evaluation and integration phase, five configurations derived from the same DeepSeek-R1-0528-Qwen3-8B parent model were evaluated on WindBench: the base model, base model + RAG, SFT model, SFT + RL model, and full model with SFT, RL, and RAG. WindBench contains 15,000 Chinese question–answer pairs across 15 task categories and is independent of the model-development resources. BLEU-4, ROUGE-1, ROUGE-2, ROUGE-L, and Text Coverage were reported on a 0–100 scale. The corresponding evaluation results are presented in From base model to agent tasks.

After integration through standardized interfaces, the finalized model was deployed in a three-path retrieval and fusion generation pipeline. In the ranking phase, the model helps reorder candidate documents. In the generation phase, the model structures responses based on evidential context and provides traceable citations. This integration enabled end-to-end connectivity between the retrieval module and the intelligent agent framework.

#### Retrieval augmented generation

In the corpus and indexing construction phase, the system integrates multi-source unstructured texts, including industry reports, technical manuals, maintenance logs, research articles, equipment specifications, and policy standards, as shown in Figure 13. All data preprocessing steps are implemented as an automated pipeline. The overall design of this pipeline is informed by existing open-source data processing frameworks, including Easy Dataset^52^ and MinerU.^53^ After data cleaning, segmentation, and format standardization, the system uses the multilingual BGE-M3 embedding model^54^ to generate paragraph-level dense semantic vectors and stores the resulting vectors in a vector database. In parallel, the system extracts entities and relationships to construct a domain-specific knowledge graph and to build keyword indices.

#### Semantic vector retrieval process

During the semantic vector retrieval process, user queries are encoded into high-dimensional vectors, and semantically relevant evidence fragments are retrieved using approximate nearest-neighbor search methods such as Faiss.^55^ This strategy enables the identification of semantically equivalent expressions despite explicit terminological differences, thereby expanding evidence coverage and improving recall robustness.

#### Graph-structured retrieval process

In the graph-structured retrieval process, the system performs multi-path reasoning based on the triple structure of the domain knowledge graph.^56^^,^^57^ For queries involving causal chains or cross-component dependencies, relevant nodes are traced along relational paths, which supports explicitly structured explanations and enhances interpretability and professional rigor.

#### Keyword retrieval process

Keyword retrieval functions as a complementary channel for rapid access to explicitly stated information, including definitions, parameters, and standard entries. When semantic or graph-based retrieval yields sparse results, keyword-matched content supplies essential contextual constraints and baseline requirements.

#### Merging, ranking, and generation phase

During the evidence merging and response generation phase, a context-aware large language model operates as a ranker within a dedicated ranking stage. It evaluates and reorders candidate evidence passages according to relevance, informational completeness, and timeliness. The highest-ranked evidence is then synthesized into structured responses. Traceability is maintained through explicit citations linking each response element to its original source.

#### Visual–physical damage assessment

The visual–physical damage assessment method integrates local surface-defect detection, global damage interpretation, and physics-based structural analysis within a unified Iwind workflow. The dataset used for the YOLO-based detector comprised 5,000 annotated wind-turbine images covering seven visible surface-defect categories: burn, crack, deformation, dirt, oil stain, peeling, and rust. The dataset was divided into training, validation, and held-out test sets at a ratio of 7:2:1, corresponding to 3,500, 1,000, and 500 images, respectively. Qwen3-VL-8B-Instruct analyzes the complete image to extract global damage attributes, including approximate fracture height, collapse direction, and overall damage morphology. Under the prescribed hazard conditions, Zwind calculates the corresponding structural loads, stress distributions, and yield-exceedance regions. The Iwind agent then integrates the YOLO detections, vision-language observations, and Zwind simulation results to generate a structured assessment report on possible structural failure types and corresponding operation and maintenance recommendations.

#### Projected blade area calculation and sensitivity analysis

To quantify the geometric contribution of blade projection under different yaw–pitch states, the projected blade area was calculated using the IEA 10-MW blade geometry adopted in the simulations. The calculation considered the spanwise blade twist distribution and the projected polygon-union area of each blade.

The original AeroDyn15 BlTwist distribution was compared with two reference configurations with uniform 0° and uniform 12° twist distributions, while other geometric parameters were kept unchanged. In addition, the projected-area difference between Yaw = +90° and Yaw = −90° was evaluated under identical blade configurations. Numerical stability was examined by comparing different blade discretization resolutions.

This analysis was used to characterize the sensitivity of geometric projection to blade configuration and yaw direction, and was considered separately from the Zwind-computed dynamic response manifold.

#### Multi-body dynamics CAE software Zwind

Developed by Zhejiang University, Zwind is a comprehensive multi-body dynamics CAE software package that facilitates the fully coupled analysis of aerodynamics, hydrodynamics, soil dynamics, structural dynamics, and servo-control systems.^58^ In this study, Zwind serves as the deterministic physics solver within the Iwind workflow. Its rigid-flexible coupled equations are formulated using the standard Kane method. As an established formulation, the Kane method provides the theoretical basis of the solver, while this section focuses on the Zwind numerical solution procedure directly relevant to the present study.

Iwind does not replace or approximate the underlying mechanics. It converts engineering requirements into solver-compatible case configurations, while Zwind assembles the generalized active and inertial contributions, solves the coupled equations of motion, and advances the structural state in time. This division preserves a clear boundary between language-level task orchestration and numerical physics. All load and structural-response results reported in this study are computed by Zwind under the prescribed model assumptions rather than inferred by the language model. The resulting outputs therefore remain traceable to a defined simulation case and can be reproduced from the corresponding solver inputs.

Specifically, Zwind supports the input of engineering wind-wave-current environments.^59^ For aerodynamics, it employs Blade Element Momentum theory and its modified methods to calculate aerodynamic forces. Hydrodynamic forces are evaluated using a hybrid time-domain method coupled with Morison equation coefficient correction.^60^ Furthermore, the software supports pile-soil interaction calculations using API p-y springs, p-y + M-θ springs,^61^ and other advanced models. Structural modeling of blades, towers, semi-submersible platforms, and anchor foundations is implemented using beam-based representations, combined with the lumped mass method or quasi-static method for mooring line simulation. For the tower analyses reported in Typhoon damage assessment, Zwind uses linear-elastic beam elements to calculate global structural responses and sectional stress demand along the tower height. Accordingly, the present analysis focuses on global beam response and sectional elastic stress demand, while local shell folding and post-yield stress redistribution require a separate nonlinear shell analysis. Finally, the platform is compatible with various servo-control algorithms.^62^^,^^63^

At each time step, Zwind evaluates the body-fixed basis vectors, partial velocities and angular velocities, and the generalized force and mass terms for each structural body. It then solves the Kane-based multibody system for the generalized coordinate accelerations and updates the generalized coordinates, velocities, and accelerations using the selected numerical integration scheme. The available schemes include the Runge-Kutta method, the Adams-Bashforth method, and the Adams-Bashforth-Moulton predictor-corrector method.

For the simulations reported here, each case is defined by the prescribed environmental loading, structural and foundation parameters, and fixed operating-state inputs such as yaw and pitch. Zwind returns deterministic load histories and structural responses for that configuration. The Iwind workflow manages case generation, execution, and result organization, but it does not modify the governing equations or replace the numerical solution produced by Zwind. This separation also allows the same deterministic solver procedure to be applied consistently to individual cases and batch yaw-pitch evaluations.

The computational workflow of the software is illustrated in Figure 14 and proceeds as follows: (1) initialize the wind turbine system at t = 0 and calculate the initial external loads from the prescribed inputs; (2) evaluate the kinematic quantities and generalized load and mass terms at time t; (3) assemble and solve the Kane-based multibody equations for the generalized coordinate accelerations; (4) advance the generalized coordinates, velocities, and accelerations to t = t + Δt; (5) calculate and output the total loads and structural responses at the specified points of interest; and (6) repeat steps (2) through (5) until the simulation is complete.

The Iwind agent interfaces with numerical solvers, including Zwind, by generating simulation input files from natural-language queries, initiating calculations, parsing output logs, and organizing the returned results under specified physical constraints.

### Quantification and statistical analysis

The quantitative evaluation of the natural-language-driven load-simulation workflow centered on task success rates. For each model configuration, we evaluated the same set of 30 predefined Zwind simulation tasks and applied binary success-or-failure criteria to both simulation-input generation and end-to-end task completion. Performance was reported as the number of successful tasks and the corresponding success rate. Workflow consistency was further assessed by comparing the maximum stress obtained from the automated workflow with that from the manually configured Zwind simulation for the same case. An absolute difference of no more than 5 MPa was used as the consistency threshold for this comparison and was intended specifically to assess agreement in workflow execution.

## Supplemental information

Video abstract. A video introducing the Iwind physics-informed LLM agent for offshore wind turbine structural analysis under typhoon and earthquake loading
